# Efficacy and safety of Shuxuening injection in intracerebral hemorrhage: a systematic review and meta-analysis

**DOI:** 10.3389/fphar.2025.1537679

**Published:** 2025-05-19

**Authors:** Wenting Song, Yaoyuan Liu, Chaofan Kang, Yazi Zhang, Xing Yan, Xinyao Jin, Yuetong Wang, Fengwen Yang, Wentai Pang

**Affiliations:** ^1^ Evidence-Based Medicine Center, Tianjin University of Traditional Chinese Medicine, Tianjin, China; ^2^ First Teaching Hospital of Tianjin University of Traditional Chinese Medicine, National Clinical Research Center for Chinese Medicine Acupuncture and Moxibustion, Tianjin, China; ^3^ School of Chinese Materia Medica, Tianjin University of Traditional Chinese Medicine, Tianjin, China

**Keywords:** Shuxuening injection, cerebral hemorrhage, intracerebral hemorrhage, systematic review, meta-analysis, randomized controlled trials

## Abstract

**Objective:**

To evaluate the efficacy and safety of Shuxuening injection (SXNI) in the treatment of patients with intracerebral hemorrhage (ICH).

**Methods:**

This study included randomized controlled trials published before 1 June 2024 in eight databases. Patients with ICH were included, with the control group receiving conventional treatment (CT) and the treatment group receiving additional SXNI on this basis. The primary outcome was neurological impairment score. The secondary outcomes were overall efficacy, cerebral hematoma volume, cerebral edema volume, activities of daily living (ADL) score, erythrocyte sedimentation rate (ESR), hematocrit (HCT), hypersensitive C-reactive protein (hs-CRP), low cut whole blood viscosity, high cut whole blood viscosity and adverse events (AE). The methodological quality of the included studies was assessed using the revised Cochrane Risk of Bias tool (ROB 2.0). For binary variables, risk ratios (RR) were calculated, while for continuous variables, mean differences (MD) or standardized mean differences (SMD) were calculated, based on 95% confidence intervals (CI).

**Results:**

A total of 29 trials involving 3,012 participants were included. Compared with the control group, the treatment group demonstrated better performance in reducing neurological impairment score [SMD = −0.99, 95% CI −1.24, −0.73], improving overall efficacy [RR = 1.22, 95% CI 1.14, 1.30] and ADL score [SMD = 2.01, 95%CI 1.55, 2.46], as well as decreasing the cerebral hematoma volume [MD = −6.98, 95% CI −8.76, −5.20] and cerebral edema volume [MD = −3.67, 95%CI -5.27, −2.06], with statistically significant differences observed. Meanwhile, the incidence of AE in the treatment group was lower than that in the control group, with a statistically significant difference [RR = 0.63, 95%CI 0.41, 0.96].

**Conclusion:**

This study indicates that the combined use of SXNI and CT may be beneficial for the treatment of patients with cerebral hemorrhage compared to the use of CT alone. However, due to the moderate to very low certainty of evidence, it is advisable to conduct highquality clinical trials to validate the findings of this study.

## 1 Introduction

Intracerebral hemorrhage (ICH), a subtype of stroke, refers to the non-traumatic rupture of cerebral vessels, leading to the accumulation of blood within the brain parenchyma. Among the various subtypes of stroke, ischemic stroke accounts for approximately 65.3% of all recorded strokes. Following ischemic stroke, cerebral hemorrhage is the second most common subtype, with about 3.4 million cases, representing 28.8% of all stroke cases. Despite the higher incidence of ischaemic stroke, it was noteworthy that the total number of disability-adjusted life-years (DALYs) attributable to ICH surpasses that of ischaemic stroke. Specifically, ICH accounts for 79.5 million DALYs, representing 49.6% of the total DALYs assigned to stroke, which was higher than the 43.8% contributed by ischemic stroke. In terms of the incidence and fatal rates, ICH has an incidence rate of 40.8 per 100,000 people and a mortality rate of 39.1 per 100,000 people (GBD, 2021 Stroke Risk Factor Collaborators et al., 2024). The high incidence and disability rates of ICH not only significantly impair patients’ health and quality of life, but also impose a substantial economic burden on society (Qiao et al., 2012; Park et al., 2018; Xiao et al., 2019).

The causes of ICH include deep perforating vasculopathy, cerebral amyloid angiopathy, cerebral arteriovenous malformation, dural arteriovenous fistula, cerebral cavernous malformation, cerebral venous thrombosis, reversible cerebral vasoconstriction syndrome, and tumors (Bushnell et al., 2024; Hilkens et al., 2024). The pathological mechanism of ICH is due to the rupture and bleeding of small arteries caused by the mass effect of the hematoma, which subsequently leads to brain damage (Keep et al., 2012). And then, bleeding activates delayed molecular mechanisms, such as microglial activation, the influx of inflammatory cells, thrombin-induced toxicity, and iron-induced toxicity (Wilkinson et al., 2018). These processes promote the breakdown of the blood-brain barrier and contribute to the development of vasogenic and cytotoxic perihematomal edema, which may manifest from a few days to several weeks following the occurrence of ICH (Cliteur et al., 2023).

Conventional treatment methods for ICH in clinical practice include antihypertensive therapy, hemostasis therapy, anticoagulation reversal, as well as surgical intervention (Hilkens et al., 2024; Li et al., 2024). These approaches aim to reduce the risk of hematoma expansion (HE) and decrease hemorrhage-related mortality in trauma patients experiencing extracranial and ICH. However, there remains controversy regarding the therapeutic efficacy and safety of drug control over hematoma expansion, as well as the potential for secondary injuries that may arise from surgical interventions (Song et al., 2024; Zhang H. et al., 2024). Moreover, current treatment options still have some limitations in terms of neuroprotective therapy, reducing blood inflammation, and improving prognosis (Cordonnier et al., 2018; Li et al., 2024).

Shuxuening injection (SXNI) is a preparation derived from *Ginkgo Biloba* extract (GBE), primarily composed of total flavonol glycosides and ginkgolides. Supplementary Material S1 contains comprehensive details about SXNI. Now, GBE is widely utilized in clinical settings as an alternative or adjunctive treatment for cardiovascular and cerebrovascular diseases (Yuan et al., 2023; Liu et al., 2024b; Zhang C. et al., 2024). Clinical studies demonstrate that SXNI was effective in preserving neural function, reducing inflammation, and facilitating the absorption of hematomas (Dong, 2012; Hu and Pu, 2015; Lu et al., 2018). This may be related to Ginkgolides may reduce blood inflammation by inhibiting the toll-like receptor 4 (TLR4) and nuclear factor κB (NF-κB) pathways (Li X. et al., 2020). It also mitigated inflammatory insults through the suppression of the CD40/NF-κB pathway and played a protective role in glutamate-induced astrocytes by regulating the Wnt and Hippo pathways (Wang et al., 2021; Li et al., 2023). And flavonoids may diminish vascular inflammatory responses by decreasing the expression of vascular cell adhesion molecule 1(VCAM-1) and intercellular adhesion molecule 1(ICAM-1), as well as reducing levels of proinflammatory factors.

The efficacy and safety of SXNI in patients with ICH are issues of significant concern to clinicians and researchers. A considerable number of clinical studies on the treatment of ICH with SXNI have been published to date, but their results vary and the quality was uneven, necessitating a systematic evidence synthesis (Zhang, 2009; Wang, 2015; Ni et al., 2018; Ma and Wang, 2019). This study utilizes the systematic review/Meta-analysis approach to assess the randomized controlled trials on SXNI for the treatment of ICH, with the aim of clarifying its efficacy and safety, and to offer guidance for clinical application.

## 2 Methods

### 2.1 Study registration and reporting guideline

The protocol for this study was registered in the International Prospective Register of Systematic Reviews (ID:CRD42024603769). This study followed the reporting guideline of the Preferred Reporting Items for Systematic Reviews and Meta-Analyses 2020 (PRISMA 2020) (Page et al., 2021).

### 2.2 Ethical statement

As this is a literature review, there is no need for ethical approval.

### 2.3 Search strategy

The two researchers independently searched four Chinese databases (CNKI, Wanfang, VIP, and SinoMed) and four English databases (PubMed, Cochrane Library, Embase, and Web of Science). The search time range was from the establishment of the database to 1 June 2024. Using a combination of subject terms and free-text terms for searching. The search query in PubMed is shown below, and the search queries for other databases can be found in Supplementary Material S2.

#1 “Cerebral Hemorrhage” [Mesh] OR “Hemorrhage, Cerebrum [Title/Abstract]” OR “Cerebrum Hemorrhage [Title/Abstract]” OR “Cerebrum Hemorrhages [Title/Abstract]” OR “Hemorrhages, Cerebrum [Title/Abstract]” OR “Cerebral Parenchymal Hemorrhage [Title/Abstract]” OR “Cerebral Parenchymal Hemorrhages [Title/Abstract]” OR “Hemorrhage, Cerebral Parenchymal [Title/Abstract]” OR “Hemorrhages, Cerebral Parenchymal [Title/Abstract]” OR “Parenchymal Hemorrhage, Cerebral [Title/Abstract]” OR “Parenchymal Hemorrhages, Cerebral [Title/Abstract]” OR “Intracerebral Hemorrhage [Title/Abstract]” OR “Hemorrhage, Intracerebral [Title/Abstract]” OR “Hemorrhages, Intracerebral [Title/Abstract]” OR “Intracerebral Hemorrhages [Title/Abstract]” OR “Hemorrhage, Cerebral [Title/Abstract]” OR “Cerebral Hemorrhages [Title/Abstract]” OR “Hemorrhages, Cerebral [Title/Abstract]” OR “Brain Hemorrhage, Cerebral [Title/Abstract]” OR “Brain Hemorrhages, Cerebral [Title/Abstract]” OR “Cerebral Brain Hemorrhage [Title/Abstract]” OR “Cerebral Brain Hemorrhage Cerebral Brain Hemorrhages [Title/Abstract]” OR “Hemorrhage, Cerebral Brain [Title/Abstract]” OR “Hemorrhages, Cerebral Brain [Title/Abstract].”

#2 “*ginkgo biloba*” [MeSH Terms] OR “*Ginkgo*” [Title/Abstract] OR “*Ginkgo biloba*” [Title/Abstract] OR “Yinxing” [Title/Abstract] OR “Yinxingye” [Title/Abstract] OR “Shuxuening” [Title/Abstract] OR “Ginaton” [Title/Abstract].

#3 Clinical Trial [Publication Type] OR randomized controlled study [Title/Abstract] OR randomized controlled trial [Title/Abstract] OR randomized study [Title/Abstract] OR randomized trial [Title/Abstract] OR randomized placebo-controlled study [Title/Abstract] OR randomized placebo-controlled trial [Title/Abstract] OR randomized placebo controlled [Title/Abstract] OR randomized placebo-controlled [Title/Abstract] OR randomized double-blind [Title/Abstract] OR randomized double blin [Title/Abstract].

#4 #1 AND #2 AND #3.

### 2.4 Eligibility criteria

#### 2.4.1 Inclusion criteria

1) Type of studies: All randomized controlled trials (RCTs) in both Chinese and English were included.
2) Participants: This study included participants who met the diagnostic criteria for ICH outlined in *the Guidelines for the Diagnosis and Treatment of Cerebral Hemorrhage in China (2019)* and *Stroke* (Chinese Society of Neurology and Chinese Stroke Society, 2019; Hilkens et al., 2024). The specific diagnostic criteria can be found in Supplementary Material S3. There were no restrictions on age, gender, race, or cause of disease.
3) Interventions and comparison: The control group received conventional treatment (including dehydrating agents, neurotrophic agents, cerebral cell activators, and symptomatic supportive therapies, such as mannitol, coenzyme A, inosine, etc.). The treatment group was treated with SXNI on the basis of conventional treatment.
4) Outcomes: The primary outcome was the neurological impairment score measured using standardized tools, including National Institute of Health Stroke Scale (NIHSS), European Stroke Scale (ESS), and China Stroke Scale (CSS). The secondary outcomes were overall efficacy, cerebral hematoma volume, cerebral edema volume, activities of daily living (ADL) score, erythrocyte sedimentation rate (ESR), hematocrit (HCT), hypersensitive C-reactive protein (hs-CRP), low cut whole blood viscosity, high cut whole blood viscosity and adverse events (AE). The specific criteria of the overall efficacy can be found in Supplementary Material S4. The outcomes included in the studies should at least encompass one of the primary or secondary outcomes.

#### 2.4.2 Exclusion criteria

1) The data were incomplete or incorrect. 2) Participants with other major diseases or those who had undergone surgical treatment were excluded from the study. 3) Studies involving other traditional Chinese medicine interventions or inconsistent conventional treatments were excluded.

### 2.5 Literature screening and data extraction

Two reviewers independently conducted literature screening and data extraction and cross-checked in strict accordance with the eligibility criteria. If there was any disagreement, the third reviewer would discuss and solve the problem. The retrieved literature was imported into NoteExpress to exclude duplicate studies, then screening the titles and abstracts of the studies, and the full text was further screened. Data extraction was performed on the final included literature, including disease name, year, sample size, course of the disease, sex, intervention, course of treatment, outcome, and adverse events. Data extraction were independently completed and verified by two researchers, and disagreements were resolved through discussion or with the assistance of a third researcher.

### 2.6 Quality assessment

The methodological quality of the included studies was assessed using the revised Cochrane Risk of Bias tool (ROB 2.0), including five areas: 1) randomization process (selection bias); 2) deviations from the intended interventions (performance bias); 3) missing outcome data (attrition bias); 4) measurement of the outcome (detection bias); 5) selection of the reported outcome (reporting bias) (Liu et al., 2021). The judgments for these domains were rated as three levels: low risk, some concerns and high risk. Finally, the overall bias was determined by summarizing the risk of bias across each domain. Risk of bias assessment were independently completed and verified by two researchers, and disagreements were resolved through discussion or with the assistance of a third researcher.

### 2.7 Statistical analysis

Statistical analysis was performed using Review Manager 5.3 and Stata 15.0. The dichotomous variables were expressed as risk ratios (RR), and the continuous variables were expressed as SMD or mean differences (MD) according to whether the measurement methods was different. The results were expressed as effect size with a 95% confidence interval (CI). Heterogeneity among studies was determined using the Chi^2^ test and reported as *I* ^*2*^. When *I* ^*2*^ was less than or equal to 50%, it was considered that the heterogeneity was low, and a fixed-effects model was used for pooling the results. Conversely, when *I* ^*2*^ was greater than 50%, it was deemed that there was high heterogeneity, subgroup analysis and sensitivity analysis were used to identify the sources of heterogeneity. If the sources of heterogeneity could not be pinpointed, a random-effects model was employed for the meta-analysis. The efficacy and safety of interventional therapy might vary significantly depending on the timing of treatment initiation in patients with ICH, and 72 h represents a critical therapeutic time point (Zhou et al., 2012; Chinese Society of Neurology and Chinese Stroke Society, 2019; Li et al., 2024). Therefore, based on the disease course, patients were divided into two subgroups: the disease course ≥72 h group and the disease course <72 h group. Additionally, subgroup analyses would also be conducted based on differences in treatment duration and SXNI dosage across the groups. Funnel plots were used to assess the publication bias.

### 2.8 Evidence certainty assessment

The certainty of evidence was assessed using the GRADE methodology, which categorizes it as high, moderate, low, or very low (Guyatt et al., 2008). Two reviewers independently evaluated the certainty of evidence for each outcome, with a third reviewer consulted to resolve any discrepancies. Detailed guidelines for assessing the certainty of evidence can be found in Supplementary Material S5.

## 3 Results

### 3.1 Literature search and screening

**FIGURE 1:**
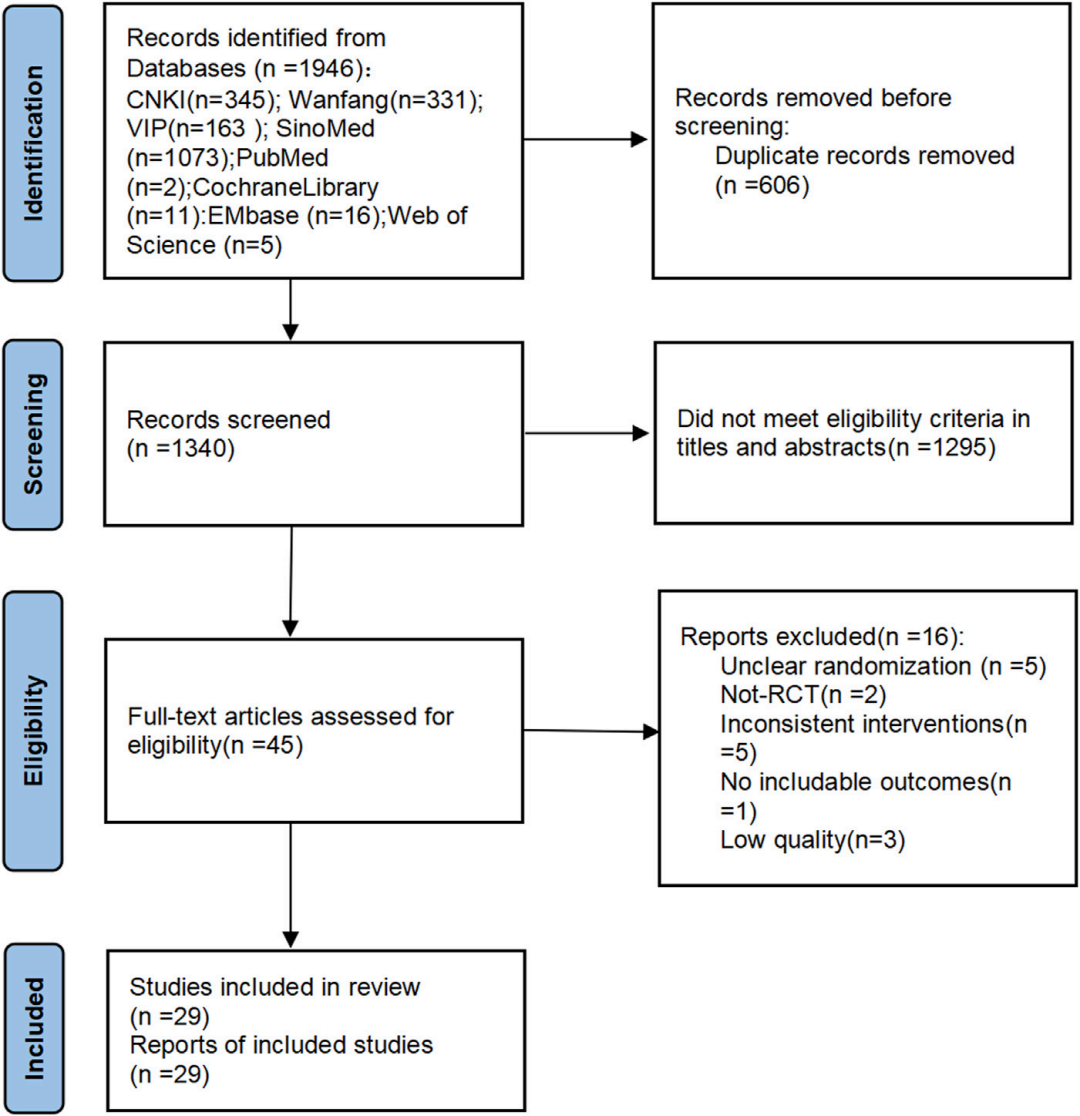
Literature screening flow and result.

Through database searches, a total of 1,946 studies were retrieved. Among them, 606 duplicate studies were excluded, and 1,340 studies were excluded because their titles and abstracts did not meet the eligibility criteria. The remaining 45 studies underwent full-text assessment. After full-text assessment, 16 studies were excluded due to the following reasons: 1) Unclear randomization (n = 5); 2) Not-RCT (n = 2); 3) Inconsistent interventions (n = 5); 4) No includable outcomes (n = 1); 5) The quality of the literature is extremely low (n = 3). Finally, 29 studies were included (Li et al., 2007; Liu, 2007; Wu, 2007; 2012; Liu and Liu, 2009; Zhang, 2009; 2011; Lan et al., 2010; Liu et al., 2010; Wang and Wang, 2010; Bai and Wang, 2012; Dong, 2012; Yuan et al., 2012; Zhang et al., 2012; 2015; Guo, 2013; Hu and Pu, 2015; Li and Hu, 2015; Wang, 2015; Wang et al., 2015; Min et al., 2016; Lu et al., 2018; Ni et al., 2018; Luo and Liu, 2019; Ma and Wang, 2019; Li, 2020; Teng, 2020; Zhong et al., 2021; Zuo et al., 2021) (Figure 1).

### 3.2 Characteristics of included studies

The total sample size of the 29 included studies was 3,012 participants, with 1,527 in the treatment group and 1,485 in the control group. These studies were published between 2007 and 2021, all in Chinese. These studies compared the efficacy and safety of SXNI combined with CT *versus* CT alone. The medications used in CT included oxiracetam injection, citicoline sodium, mannitol, edaravone injection, GM1, glycerol and fructose, furosemide, coenzyme A, inosine, β-aescin sodium. The average age of the patients ranged from 51.00 to 69.12, and the course of treatment ranged from 1 to 3 weeks. In the majority of the trials, males outnumbered females. No significant disparities were observed in the baseline characteristics across all the studies. The specific characteristics are shown in Table 1.

**TABLE 1:** Characteristics of included studies.

Study	Disease	Year (T/C)	Sample size (T/C)	Course of the disease (T/C)	Sex (M/F)		Intervention		Course of treatment (days)	Outcome
					T	C	T	C		
Bai and Wang (2012)	HICH	63.70 ± 7.38/62.9 ± 8.56	54/54	(7–8) d	29/25	28/26	SXNI (10 mL)+C	CT	10–14d	②⑪
Dong (2012)	HICH	40–70	56/56	-	-	-	SXNI (10 mL)+C	CT	20d	③
Guo (2013)	HICH	40–78	41/41	≥7d	-	-	SXNI (20 mL)+C	CT	14d	③⑪
Hu and Pu (2015)	ICH	-	61/61	-	-	-	SXNI (20 mL)+C	CT (bdij)	7d	②⑥⑦⑨⑩
Lan et al. (2010)	HICH	50–75	32/32	(24–72) h	-	-	SXNI (10 mL)+C	CT	21d	③
Li and Hu (2015)	ICH	54.5 ± 3.5/54.1 ± 3.2	42/42	<24 h	25/17	26/16	SXNI (10 mL)+C	CT (bdij)	7d	②⑥⑦⑨⑩
Li et al. (2007)	ICH	50–75	31/31	(24–72) h	-	-	SXNI (10 mL)+C	CT	21d	①③
Li (2020)	AICH	68.29 ± 5.77/68.25 ± 5.74	35/35	(3.52 ± 0.68) h/(3.55 ± 0.71) h	18/17	20/15	SXNI(5 mL)+C	CT (a)	14d	①⑤
Liu and Liu (2009)	HICH	62.15 ± 7.21/63.03 ± 8.16	32/40	acute phase	18/14	25/15	SXNI (10 mL)+C	CT	14d	①②③
Liu et al. (2010)	HICH	63.44 ± 7.38/62.30 ± 8.56	35/38	acute phase	20/15	24/14	SXNI (10 mL)+C	CT	14d	①②③
Liu (2007)	HICH	51 ± 3.6/52 ± 4.1	27/29	≥10d	19/8	21/8	SXNI (15 mL)+C	CT (bcdj)	15d	①③④
Lu et al. (2018)	HICH	56.82 ± 18.36/57.44 ± 20.38	26/26	10d	-	-	SXNI (20 mL)+C	CT	21d	①②③④⑪
Luo and Liu (2019)	ICH	54.5 ± 12.5/59.5 ± 14.5	46/46	(24–72) h	30/16	22/24	SXNI(−)+C	CT (de)	14d	①③④
Ma and Wang (2019)	AICH	63–81	105/105	<24 h	-	-	SXNI(5 mL)+C	CT (a)	14d	①②③④⑤⑪
Min et al. (2016)	AICH	61.55 ± 11.47/62.52 ± 10.30	41/41	(13.29 ± 10.28) h/(13.76 ± 10.12) h	31/10	30/11	SXNI(5 mL)+C	CT (e)	14d	①②③④⑪
Ni et al. (2018)	AICH	58.97 ± 4.51/59.27 ± 4.62	49/49	(18.59 ± 2.56) h/(18.97 ± 2.70) h	30/19	28/21	SXNI(20 mL)+C	CT	14d	①②③⑤⑧⑪
Teng (2020)	AICH	69.12 ± 4.30/67.36 ± 3.24	39/39	(12.95 ± 6.73) h/(13.06 ± 8.47) h	22/17	20/19	SXNI(5 mL)+C	CT (e)	14d	①②③④⑤⑧⑪
Wang and Wang (2010)	HICH	55 ± 15/56 ± 12	80/80	(48–72) h	42/38	40/40	SXNI (10 mL)+C	CT (dgh)	20d	②⑪
Wang et al. (2015)	HICH	63.75/62.38	34/34	<48 h	19/15	20/14	SXNI(20 mL)+C	CT (dh)	14d	③④
Wang (2015)	ICH	55.1 ± 0.2/54.5 ± 1.1	122/122	(24–72) h	64/58	62/60	SXNI (10 mL)+C	CT (bdij)	7d	②⑥⑦⑨⑩
Wu (2007)	ICH	60.2 ± 15.3/60.6 ± 16.5	102/53	(24–72) h	53/49	28/25	SXNI (10 mL)+C	CT	21d	①③
Wu (2012)	HICH	60.3 ± 8.4/61.0 ± 8.9	62/62	5d	37/25	35/27	SXNI (10 mL)+C	CT (b)	14d	②③④
Yuan et al. (2012)	AICH	39–82/41–83	45/43	<48 h	24/21	22/21	SXNI(20 mL)+C	CT	14d	①②③
Zhang et al. (2012)	AICH	62.12 ± 6.32/59.25 ± 6.31	40/40	(1.5–32.5) h/(2–33) h	25/15	22/18	SXNI (5 mL)+C	CT (f)	14d	①②⑪
Zhang et al. (2015)	ICH	45–78	122/122	-	-	-	SXNI (10 mL)+C	CT (bdij)	7d	②⑥⑦⑨⑩
Zhang (2009)	ICH	60/61	46/40	recovery period	36/10	29/11	SXNI(20 mL)+C	CT (d)	15d	①③
Zhang (2011)	ICH	58.5 ± 10.8/57.5 ± 11.2	29/31	(1–5) d	20/9	22/9	SXNI (20 mL)+C	CT	14d	②
Zhong et al. (2021)	AICH	60.16 ± 2.61/60.13 ± 2.57	43/43	(10.15 ± 1.51) h/(10.12 ± 1.53) h	30/13	29/14	SXNI(5 mL)+C	CT (e)	14d	①⑧⑪
Zuo et al. (2021)	AICH	59.16 ± 6.74/60.02 ± 5.97	50/50	(19.23 ± 7.17) h/(20.11 ± 6.89) h	29/21	29/21	SXNI(20 mL)+C	CT	14d	①②③⑤⑧⑪

Abbreviations: T, treatment group; C, control group; CT, conventional treatment; HICH, hypertensive intracerebral hemorrhage; AICH, acute intracerebral hemorrhape; ICH, intracerebral hemorrhage; SXNI, shuxuening injection; a, Oxiracetam Injection; b, Citicoline Sodium; c, β-aescin sodium; d, Mannitol; e, Edaravone Injection; f, GM1; g, Glycerol and Fructose; h, Furosemide; i, Coenzyme A; j, Inosine; ① neurological impairment score; ② overall efficacy; ③ cerebral hematoma volume; ④ cerebral edema volume; ⑤ activities of daily living score; ⑥ erythrocyte sedimentation rate; ⑦ hematocrit; ⑧ hypersensitive C-reactive protein; ⑨ low cut whole blood viscosity; ⑩ high cut whole blood viscosity; ⑪ adverse events.

### 3.3 Risk of bias assessment

**FIGURE 2:**
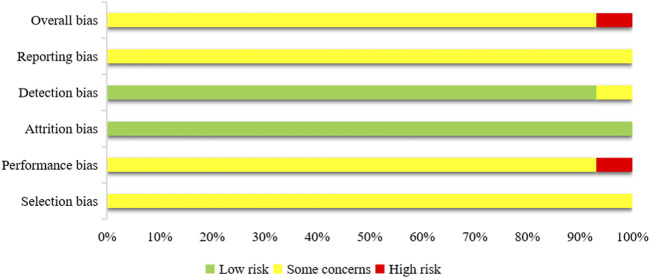
Risk of bias result of included studies.

The 29 studies were assessed based on the five domains of ROB 2.0. In terms of the randomization process, 29 studies reported that the order of allocation was randomized, baseline balanced between groups, and were rated as some concern. Regarding deviations from intended interventions, the outcomes of 27 studies were not easily affected by the research environment and were rated as some concerns; 2 studies (Wang and Wang, 2010; Zhang et al., 2012) had outcomes that were more susceptible to bias due to environmental influences, which were rated as high risk. In terms of missing outcome data, none of the 29 studies reported any dropout cases, and all outcome data were complete, which was rated as low risk. For outcome measurement, although 27 studies did not describe blinding of assessors, their outcomes were not easily influenced by measurement, which was rated as low risk; 2 studies (Wang and Wang, 2010; Zhang et al., 2012) had outcomes that might be somewhat influenced by measurement, which was rated as some concerns. In terms of selective reporting of results, although none of the 29 studies had a predetermined plan, none of them reported selectively, which was rated as some concerns. Among the overall risk of bias, 27 studies were rated as some concerns, and 2 studies (Wang and Wang, 2010; Zhang et al., 2012) were rated as high risk. The results of the bias risk assessment for the included studies are shown in Figure 2.

### 3.4 Primary outcome

#### 3.4.1 Neurological impairment score

**FIGURE 3:**
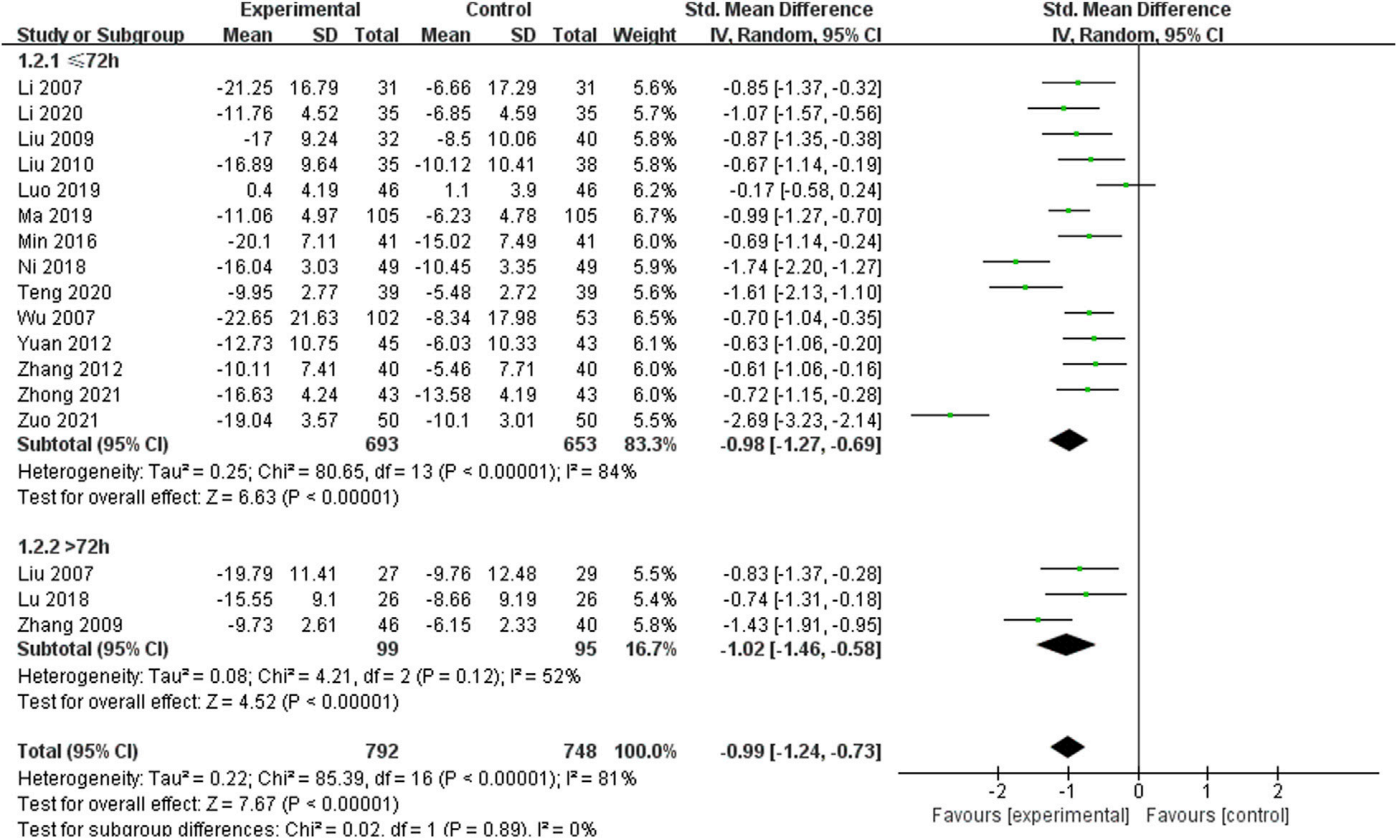
Meta-analysis results of the effect of Shuxuening injection combined with conventional treatment vs conventional treatment on neurological impairment score.

Seventeen studies including 1,540 patients reported the change in neurological impairment score, with high heterogeneity among the studies (*I* ^*2*^ = 81%) (Li et al., 2007; Liu, 2007; Wu, 2007; Liu and Liu, 2009; Zhang, 2009; Liu et al., 2010; Yuan et al., 2012; Zhang et al., 2012; Min et al., 2016; Lu et al., 2018; Ni et al., 2018; Luo and Liu, 2019; Ma and Wang, 2019; Li, 2020; Teng, 2020; Zhong et al., 2021; Zuo et al., 2021) (Figure 3). The results of the sensitivity analysis indicated that excluding any single study did not reduce the significant heterogeneity of the pooled results. Therefore, a random-effects model was used to pool the results. Subgroup analysis was conducted based on the course of disease (≤72 h or >72 h). In the ≤72 h subgroup (14 RCTs including 1,346 participants), SXNI combined with CT was more effective than CT alone in reducing neurological impairment score [SMD = −0.98, 95%CI (−1.27, −0.69), *p* < 0.00001]. In the >72 h subgroup (3 RCTs including 194 participants), SXNI combined with CT was more effective than CT alone in reducing neurological impairment score [SMD = −1.02, 95%CI (−1.46, −0.58), *p* = 0.12]. No statistically significant difference was observed between the two subgroups (*p* = 0.89).

### 3.5 Secondary outcomes

#### 3.5.1 Overall efficacy

**FIGURE 4:**
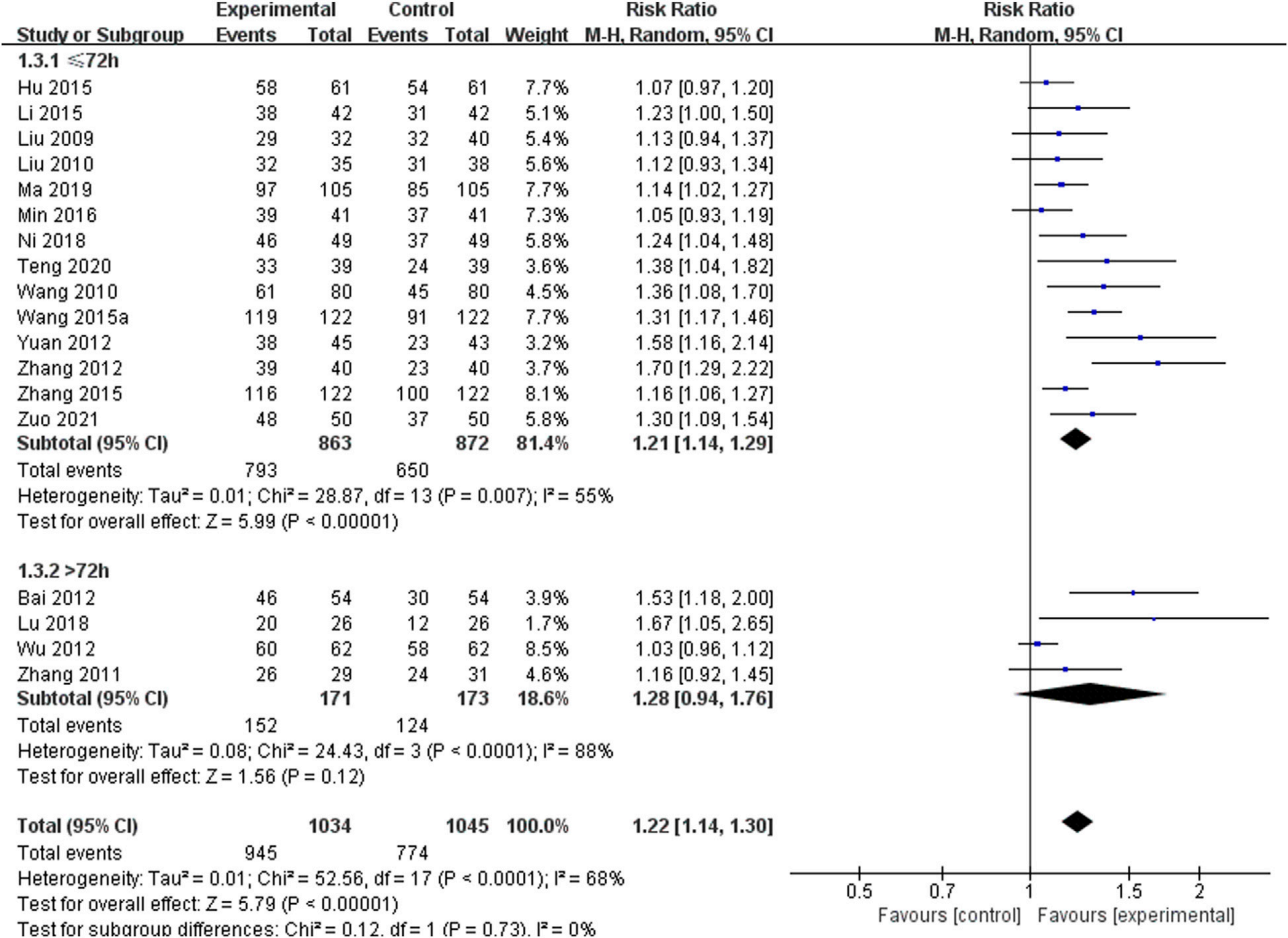
Meta-analysis results of the effect of Shuxuening injection combined with conventional treatment vs. conventional treatment on overall efficacy.

A total of 18 studies including 2,079 patients reported overall efficacy, with high heterogeneity among the studies (*I* ^*2*^ = 68%) (Liu and Liu, 2009; Liu et al., 2010; Wang and Wang, 2010; Zhang, 2011; Bai and Wang, 2012; Wu, 2012; Yuan et al., 2012; Zhang et al., 2012; 2015; Hu and Pu, 2015; Li and Hu, 2015; Wang, 2015; Min et al., 2016; Lu et al., 2018; Ni et al., 2018; Ma and Wang, 2019; Teng, 2020; Zuo et al., 2021) (Figure 4). The results of the sensitivity analysis indicated that excluding any single study did not reduce the significant heterogeneity of the pooled results. Therefore, a random-effects model was used to pool the results. Subgroup analysis was conducted based on the course of the disease (≤72 h or >72 h). In the ≤72 h subgroup (14 RCTs including 1,735 participants), SXNI combined with CT was more effective than CT alone in improving overall efficacy [RR = 1.21, 95% CI (1.14, 1.29), *p* = 0.007]. In the >72 h subgroup (4 RCTs including 344 participants), there was no difference in overall efficacy between the SXNI combined with CT and CT alone [RR = 1.28, 95% CI (0.94, 1.76), *p* < 0.0001]. No statistically significant difference was observed between the two subgroups (*p* = 0.73).

#### 3.5.2 Cerebral hematoma volume

**FIGURE 5:**
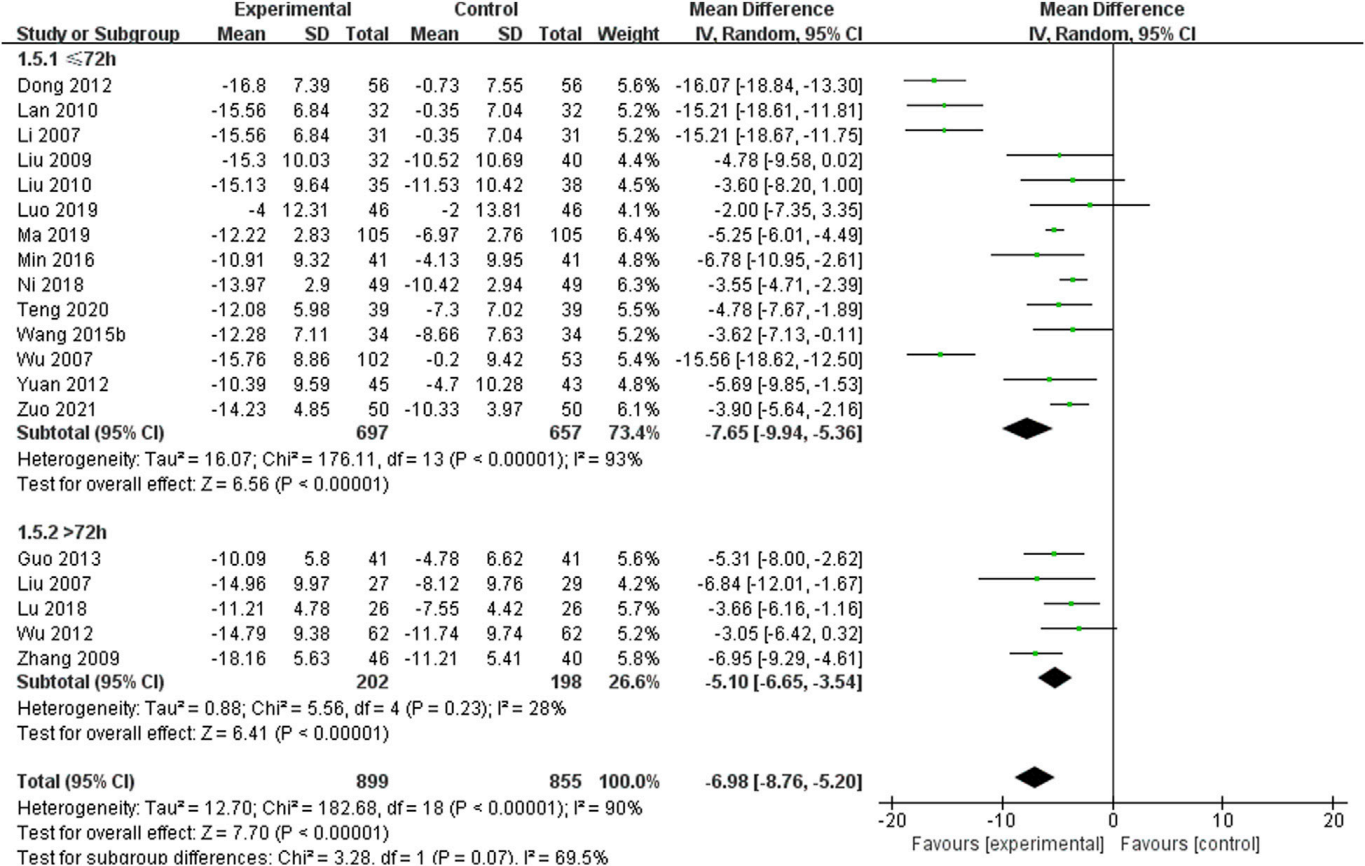
Meta-analysis results of the effect of Shuxuening injection combined with conventional treatment vs conventional treatment on cerebral hematoma volume.

A total of 19 studies including 1,754 patients reported the cerebral hematoma volume, with high heterogeneity among the studies (*I* ^*2*^ = 90%) (Li et al., 2007; Liu, 2007; Wu, 2007; 2012; Liu and Liu, 2009; Zhang, 2009; Lan et al., 2010; Liu et al., 2010; Dong, 2012; Yuan et al., 2012; Guo, 2013; Wang et al., 2015; Min et al., 2016; Lu et al., 2018; Ni et al., 2018; Luo and Liu, 2019; Ma and Wang, 2019; Teng, 2020; Zuo et al., 2021) (Figure 5). The results of the sensitivity analysis indicated that excluding any single study did not reduce the significant heterogeneity of the pooled results. Therefore, a random-effects model was used to pool the results. Subgroup analysis was conducted based on the course of disease (≤72 h or >72 h). In the ≤72 h subgroup (14 RCTs including 1,354 participants), SXNI combined with CT was more effective than CT alone in reducing the cerebral hematoma volume [MD = −7.65, 95% CI (−9.94, −5.36), *p* < 0.00001]. In the >72 h subgroup (5 RCTs including 400 participants), SXNI combined with CT was more effective than CT alone in reducing the cerebral hematoma volume [MD = −5.10, 95% CI (−6.65, −3.54), *p* = 0.23]. No statistically significant difference was observed between the two subgroups (*p* = 0.07).

#### 3.5.3 Cerebral edema volume

**FIGURE 6:**
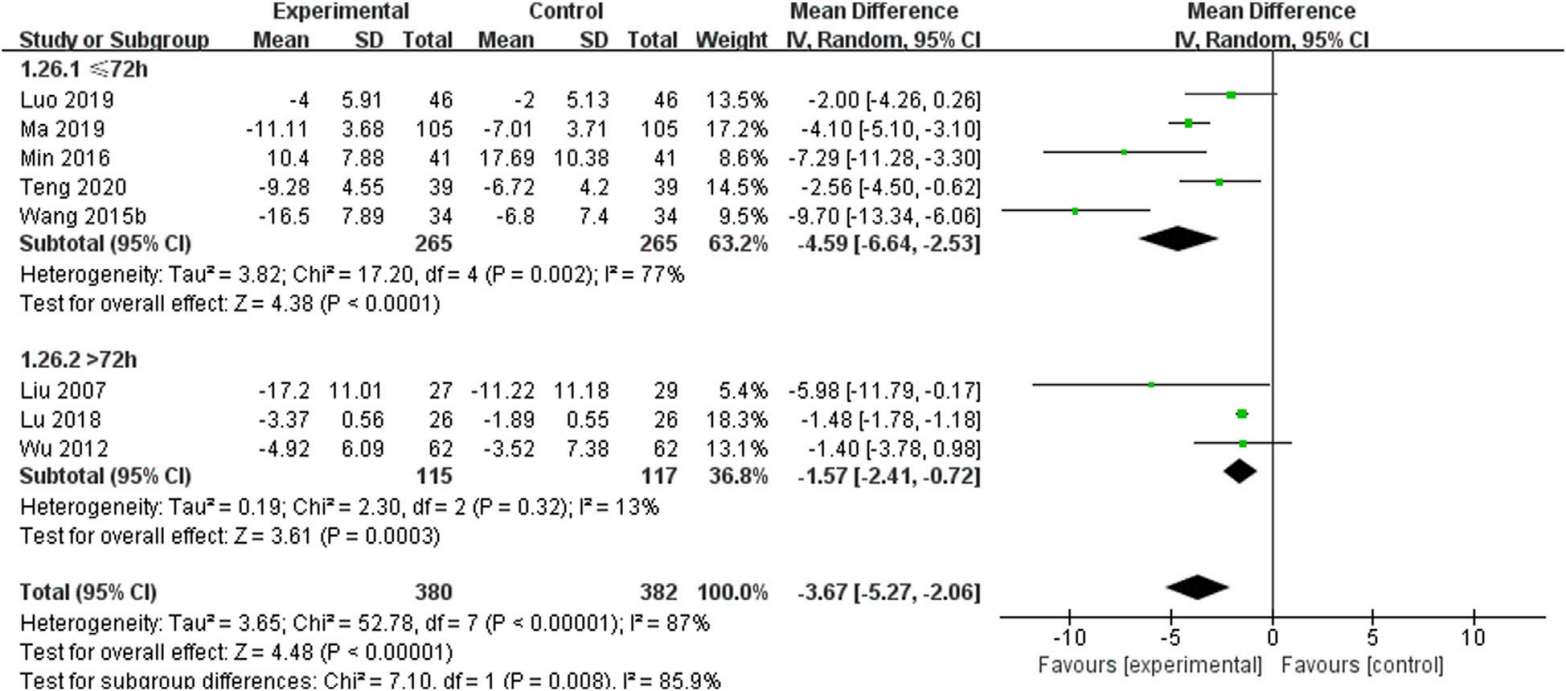
Meta-analysis results of the effect of Shuxuening injection combined with conventional treatment vs conventional treatment on cerebral edema volume.

A total of 8 studies including 762 patients reported the cerebral edema volume, with high heterogeneity among the studies (*I* ^*2*^ = 87%) (Liu, 2007; Wu, 2012; Wang et al., 2015; Min et al., 2016; Lu et al., 2018; Luo and Liu, 2019; Ma and Wang, 2019; Teng, 2020) (Figure 6). The results of the sensitivity analysis indicated that excluding any single study did not reduce the significant heterogeneity of the pooled results. Therefore, a random-effects model was used to pool the results. Subgroup analysis was conducted based on the course of disease (≤72 h or >72 h). In the >72 h subgroup (5 RCTs including 530 participants), SXNI combined with CT was more effective than CT alone in reducing the cerebral edema volume [MD = −4.59, 95% CI (−6.64, −2.53), *p* = 0.002]. In the >72 h subgroup (3 RCTs including 232 participants), SXNI combined with CT was more effective than CT alone in reducing the cerebral edema volume [MD = −1.57, 95% CI (−2.41, −0.72), *p* = 0.32]. The comparison between subgroups showed significantly better efficacy in the <72 h subgroup than in the >72 h subgroup (*p* = 0.008).

#### 3.5.4 Activities of daily living score

**FIGURE 7:**
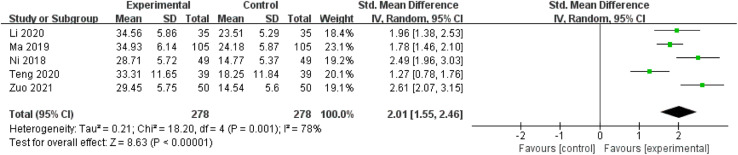
Meta-analysis results of the effect of Shuxuening injection combined with conventional treatment vs conventional treatment on Activities of daily living score.

Five studies including 556 patients reported the ADL score for patients with disease course ≤72h, showing high heterogeneity among the studies (I^2^ = 78%) (Ni et al., 2018; Ma and Wang, 2019; Li, 2020; Teng, 2020; Zuo et al., 2021) (Figure 7). The results of the sensitivity analysis indicated that excluding any single study did not reduce the significant heterogeneity of the pooled results. Therefore, a random-effects model was used to pool the results. The results showed that the difference between the two groups was statistically significant [SMD = 2.01, 95%CI (1.55, 2.46), *p =* 0.001]. It may be concluded that SXNI combined with CT has a better effect on improving the ADL score than CT alone.

#### 3.5.5 Erythrocyte sedimentation rate

**FIGURE 8:**
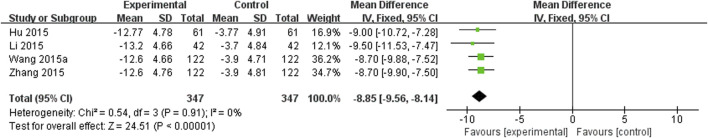
Meta-analysis results of the effect of Shuxuening injection combined with conventional treatment vs conventional treatment on erythrocyte sedimentation rate.

Four studies including 694 patients reported the ESR for patients with disease course ≤72 h (Hu and Pu, 2015; Li and Hu, 2015; Wang, 2015; Zhang et al., 2015) (Figure 8). Due to low heterogeneity between studies (I^2^ = 0%), a fixed-effects model was used for analysis. The results showed the difference between the two groups was statistically significant [MD = −8.85, 95%CI (−9.56, −8.14), *p* = 0.91]. The ESR of SXNI combined with CT was lower than that of CT alone.

#### 3.5.6 Hematocrit

**FIGURE 9:**
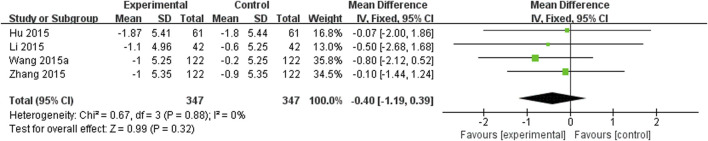
Meta-analysis results of the effect of Shuxuening injection combined with conventional treatment vs conventional treatment on hematocrit.

Four studies including 694 patients reported the HCT for patients with disease course ≤72 h (Hu and Pu, 2015; Li and Hu, 2015; Wang, 2015; Zhang et al., 2015) (Figure 9). Due to low heterogeneity between studies (*I* ^*2*^ = 0%), a fixed-effects model was used for analysis. The results showed that there was no difference in HCT between the SXNI combined with CT and CT alone [MD = −0.40, 95% CI (−1.19, 0.39), *p* = 0.88].

#### 3.5.7 Hypersensitive C-reactive protein

**FIGURE 10:**
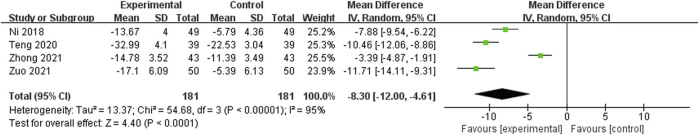
Meta-analysis results of the effect of Shuxuening injection combined with conventional treatment vs conventional treatment on hypersensitive C-reactive protein.

Four studies including 362 patients reported the hs-CRP for patients with disease course ≤72h, showing high heterogeneity among the studies (*I* ^*2*^ = 95%) (Ni et al., 2018; Teng, 2020; Zhong et al., 2021; Zuo et al., 2021) (Figure 10). The results of the sensitivity analysis indicated that excluding any single study did not reduce the significant heterogeneity of the pooled results. Therefore, a random-effects model was used to pool the results. The results showed that the difference between the two groups was statistically significant [MD = −8.30, 95%CI (−12.00, −4.61), *p* < 0.00001]. It may be concluded that SXNI combined with CT has a better effect on reducing the hs-CRP than CT alone.

#### 3.5.8 Low cut whole blood viscosity

**FIGURE 11:**
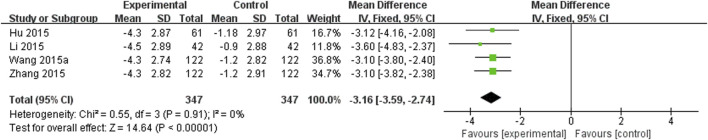
Meta-analysis results of the effect of Shuxuening injection combined with conventional treatment vs conventional treatment on low cut whole blood viscosity.

Four studies including 694 patients reported the low cut whole blood viscosity for patients with disease course ≤72 h (Hu and Pu, 2015; Li and Hu, 2015; Wang, 2015; Zhang et al., 2015) (Figure 11). Due to low heterogeneity between studies (*I* ^*2*^ = 0%), a fixed-effects model was used for analysis. The results showed the difference between the two groups was statistically significant [MD = −3.16, 95%CI (−3.59, −2.74), *p* = 0.91]. The low cut whole blood viscosity of SXNI combined with CT was lower than that of CT alone.

#### 3.5.9 High cut whole blood viscosity

**FIGURE 12:**
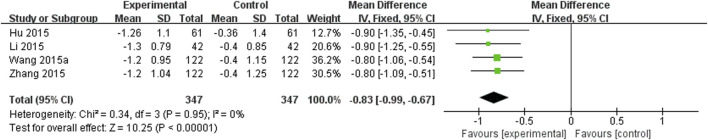
Meta-analysis results of the effect of Shuxuening injection combined with conventional treatment vs conventional treatment on high cut whole blood viscosity.

Four studies including 694 patients reported the high cut whole blood viscosity for patients with disease course ≤72 h (Hu and Pu, 2015; Li and Hu, 2015; Wang, 2015; Zhang et al., 2015) (Figure 12). Low heterogeneity was observed among these studies (*I* ^*2*^ = 0%). Accordingly, the fixed effect model was used for analysis. The results showed the difference between the two groups was statistically significant [MD = −0.83, 95% CI (−0.99, −0.67), *p* = 0.95], it combined use of SXNI and CT demonstrated a greater efficacy in reducing high cut whole blood viscosity compared to the use of CT alone.

#### 3.5.10 Adverse events

**FIGURE 13:**
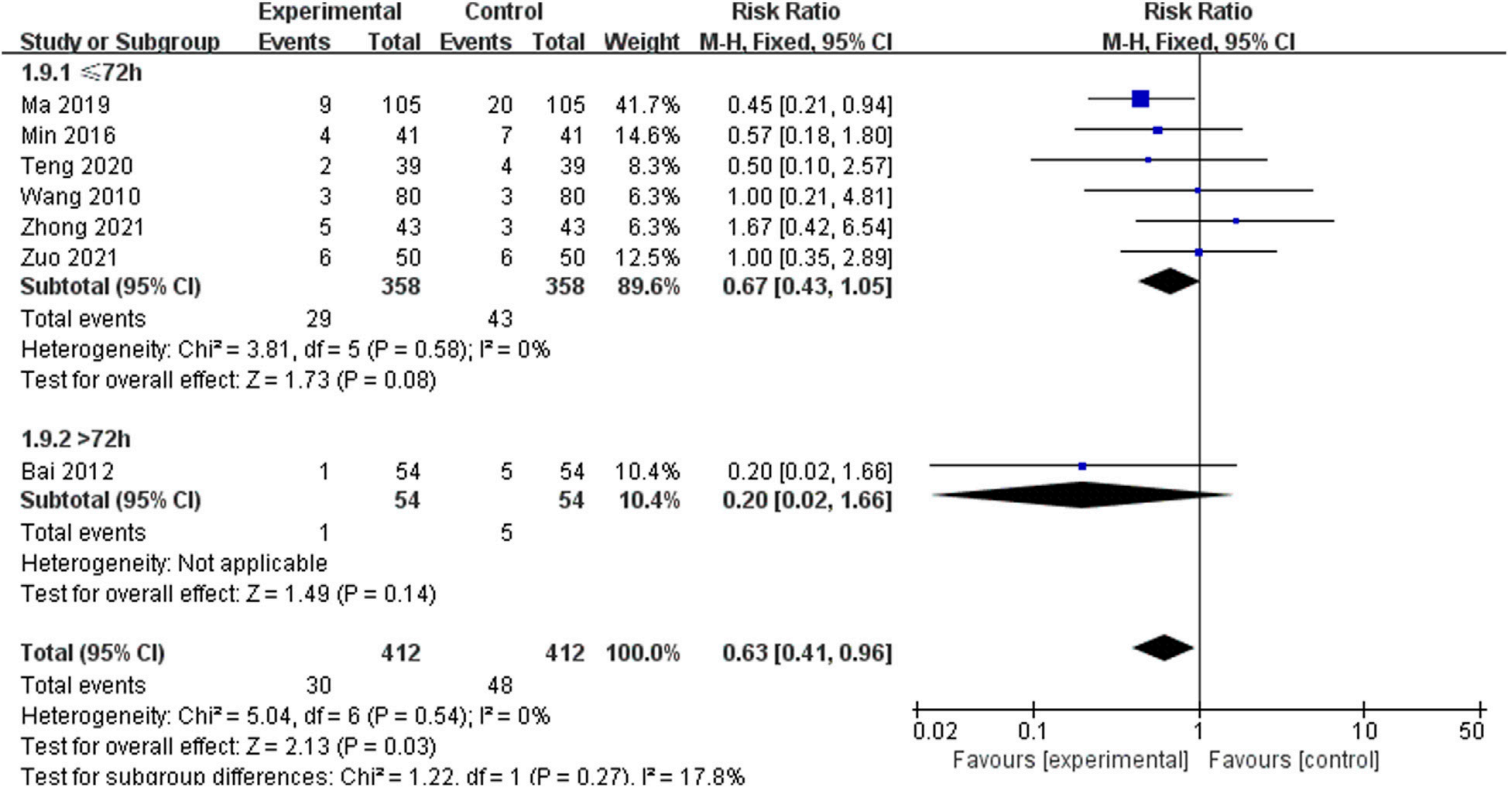
Meta-analysis results of the effect of Shuxuening injection combined with conventional treatment vs conventional treatment on adverse events.

A total of 11 studies including 1,136 patients reported data on specific AE (Wang and Wang, 2010; Bai and Wang, 2012; Zhang et al., 2012; Guo, 2013; Min et al., 2016; Lu et al., 2018; Ni et al., 2018; Ma and Wang, 2019; Teng, 2020; Zhong et al., 2021; Zuo et al., 2021). Of which four studies clearly stated that no AE occurred in either group (Zhang et al., 2012; Guo, 2013; Lu et al., 2018; Ni et al., 2018) (Table 2). Meta-analysis was performed for the other 7 studies (Wang and Wang, 2010; Bai and Wang, 2012; Min et al., 2016; Ma and Wang, 2019; Teng, 2020; Zhong et al., 2021; Zuo et al., 2021) (Figure 13). Due to low heterogeneity between studies (*I* ^*2*^ = 0%), a fixed-effects model was used for analysis. In the >72 h subgroup (6 RCTs including 716 participants), there was no difference in AE between the SXNI combined with CT and CT alone [RR = 0.67, 95% CI (0.43, 1.05), *p* = 0.58]. In the >72 h subgroup (1 RCTs including 108 participants), there was no difference in AE between the SXNI combined with CT and CT alone [RR = 0.20, 95% CI (0.02, 1.66), *p* = 0.14]. In the overall result (7 RCTs including 824 participants), the meta-analysis showed that the incidence of adverse events in the SXNI combined with CT was lower than CT alone [RR = 0.63, 95% CI (0.41, 0.96), *p* = 0.03]. No statistically significant difference was observed between the two subgroup (*p* = 0.27).

**TABLE 2:** Incidence of adverse events.

Study	Sample size		Adverse events	
	T	C	T	C
Wang 2010	80	80	3 (deterioration)	3 (deterioration)
Bai 2012	54	54	1 (intracranial hematoma enlargement)	5 (intracranial hematoma enlargement)
Ma 2019	105	105	9 (3 nausea and vomiting, 2 dizziness, 3 diarrhea, 1 allergy)	20 (4 nausea and vomiting, 7 dizziness, 6 diarrhea, 3 allergy)
Zhong 2021	43	43	5 (4 headache, 1 blood pressure decrease)	3 (2 rash, 1 headache)
Teng 2020	39	39	2 (1 rash, 1 pruritus)	4 (1 rash, 1 pruritus, 2 injection site swelling)
Zuo 2021	50	50	6 (2 allergy, 1 elevated blood pressure, 2 increased white blood cell count, 1 abnormal liver function)	6 (1 allergy, 2 elevated blood pressure, 1 increased white blood cell count, 2 abnormal liver function)
Min 2016	41	41	4 (3 allergy, 1 abnormal liver function)	7 (2 allergy, 2 elevated blood pressure, 1 increased white blood cell count, 2 abnormal liver function)

### 3.6 Sensitivity analyses

By successively excluding literature, the combined results did not change significantly, indicating good stability of the outcomes. However, it was not possible to reduce the heterogeneity of the combined results by excluding literature. The graph of sensitivity analysis may be found in Supplementary Material S6.

### 3.7 Subgroup analyses

Subgroup analysis was conducted based on different SXNI doses and durations of treatment. The low-dose subgroup was defined as those who received 10 mL or less of SXNI, while the high-dose subgroup received more than 10 mL. For the duration of treatment, the short-term treatment subgroup was defined as those treated for 14 days or less, and the long-term treatment subgroup was defined as those treated for more than 14 days. The results of each subgroup analysis were consistent with the overall results (Tables 3, 4). For patients with disease course ≤72h, the long-term treatment group was superior to the short-term treatment group in alleviating cerebral hematoma volume (*p*_interaction_ < 0.00001). In terms of reducing the cerebral hematoma volume, the low-dose subgroup outperformed the high-dose subgroup (*p*_interaction_ = 0.002). In terms of reducing cerebral edema volume, the high-dose subgroup was superior to the low-dose subgroup (*p*_interaction_ = 0.006). In improving ADL scores, the high-dose subgroup was superior to the low-dose subgroup (*p*_interaction_ = 0.001).

**TABLE 3:** Subgroup analysis of the ≤72 h disease course group.

Subgroup		No.S	MD/SMD/RR	95%CI	*I* ^*2*^	*P*_interaction_
Different treatment duration						
Neurological impairment score	short term	12	−1.02	−1.36 to −0.68	86	0.21
	long term	2	−0.74	−1.03 to −0.46	0	
Overall efficacy	short term	13	1.22	1.17 to 1.28	55	0.39
	long term	1	1.36	1.08 to 1.70	—	
Cerebral hematoma volume	short term	10	−4.59	−5.19 to −3.99	4	<0.00001
	long term	4	−15.58	−17.14 to −14.01	0	
Adverse events	short term	5	0.65	0.41 to 1.04	0	0.61
	long term	1	1.00	0.21 to 4.81	—	
Different dosages of SXNI						
Neurological impairment score	low dose	10	−0.86	−1.03 to −0.70	32	0.17
	high dose	3	−1.67	−2.83 to −0.52	94	
Overall efficacy	low dose	10	1.23	1.17 to 1.29	53	0.77
	high dose	4	1.25	1.14 to 1.36	70	
Cerebral hematoma volume	low dose	9	−9.77	−13.56 to −5.98	94	0.002
	high dose	4	−3.75	−4.66 to −2.85	0	
Cerebral edema volume	low dose	3	−4.04	−5.82 to −2.26	58	0.006
	high dose	1	−9.70	−13.34 to −6.06	—	
Activities of daily living score	low dose	3	1.67	1.30 to 2.04	50	0.001
	high dose	2	2.55	1.17 to 2.93	0	
Erythrocyte sedimentation rate	low dose	3	−8.82	−9.59 to −8.04	0	0.85
	high dose	1	−9	−10.72 to −7.28	—	
Hematocrit	low dose	3	−0.46	−1.33 to 0.40	0	0.71
	high dose	1	−0.07	−2.00 to 1.86	—	
Hypersensitive C-reactive protein	low dose	2	−6.92	−13.85 to 0.01	98	0.49
	high dose	2	−9.69	−13.44 to −5.95	85	
Low cut whole blood viscosity	low dose	3	−3.17	−3.63 to −2.71	0	0.93
	high dose	1	−3.12	−4.16 to −2.08	—	
High cut whole blood viscosity	low dose	3	−0.82	−0.99 to −0.65	0	0.75
	high dose	1	−0.9	−1.35 to −0.45	—	
Adverse events	low dose	5	0.62	0.38 to 1.02	0	0.43
	high dose	1	1.00	0.35 to 2.89	—	

Abbreviations, No.S, numbers of studies; MD, mean differences; SMD, standardized mean differences; RR, risk ratio; CI, confidence interval; *P*_interaction_, *P* for interaction.

**TABLE 4:** Subgroup analysis of the >72 h disease course group.

Subgroup		No.S	MD/SMD/RR	95%CI	*I* ^*2*^	*P*_interaction_
Different treatment duration						
Overall efficacy	short term	3	1.21	0.89 to 1.64	88	0.26
	long term	1	1.67	1.05 to 2.65	—	
Cerebral hematoma volume	short term	2	−4.41	−6.58 to −2.25	5	0.47
	long term	3	−5.60	−8.02 to −3.18	48	
Cerebral edema volume	short term	1	−1.40	−3.78 to 0.98	0	0.94
	long term	2	−1.49	−1.79 to −1.19	56	
Different dosages of SXNI						
Overall efficacy	low dose	2	1.25	0.71 to 2.21	94	0.86
	high dose	2	1.32	0.90 to 1.94	57	
Cerebral hematoma volume	low dose	1	−3.05	−6.42 to 0.32	—	0.20
	high dose	4	−5.50	−7.11 to −3.88	22	
Cerebral edema volume	low dose	1	−1.40	−3.78 to 0.98	0	0.94
	high dose	2	−1.49	−1.79 to −1.19	56	

Abbreviations: No.S, numbers of studies; MD, mean differences; SMD, standardized mean differences; RR, risk ratio; CI, confidence interval; *P*_interaction_, *P* for interaction.

### 3.8 Publication bias

**FIGURE 14:**
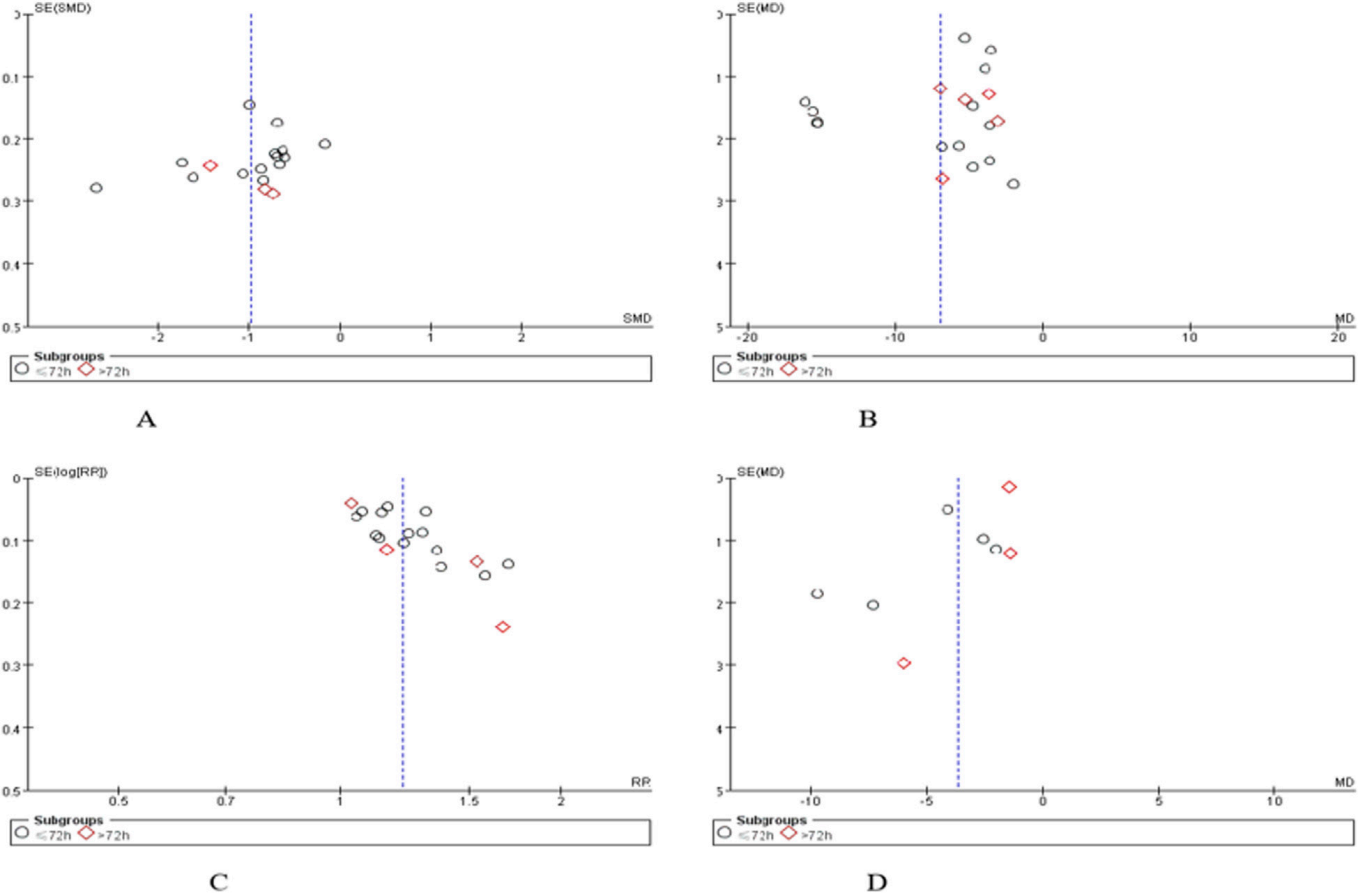
Publication bias assessed by funnel plots. **(A)** neurological impairment score, **(B)** cerebral hematoma volume, **(C)** overall efficacy, **(D)** cerebral edema volume.

Funnel plots were used to assess publication bias for the four outcomes: neurological impairment score, cerebral hematoma volume, overall efficacy and cerebral edema volume. The funnel plots of neurological deficit score (Figure 14A), cerebral hematoma volume (Figure 14B), overall efficacy (Figure 14C) and cerebral edema volume (Figure 14D) showed obvious asymmetry, suggesting that there may be publication bias in the included studies.

### 3.9 Certainty of evidence

Table 5 shows the assessment of the certainty of evidence. According to the results, the evidence for ESR, low cut whole blood viscosity, high cut whole blood viscosity, AE were considered to moderate certainty; HCT was considered to low certainty; neurological impairment score, overall efficacy, cerebral hematoma volume, cerebral edema volume, ADL score, and hs-CRP were considered as very low certainty.

**TABLE 5:** Certainty of evidence.

Outcomes	Certainty assessment							No. of participants		Effect		Certainty
	No.S	Study design	Risk of bias	Inconsistency	Indirectness	Imprecision	Publication bias	Treatment group	Control group	Relative (95% CI)	Absolute (95% CI)	
Neurological impairment score	17	RCT	serious	very serious	not serious	not serious	serious	792	748	N/A	SMD -0.99 (−1.24 to −0.73)	⊕○○○○○ VERY LOW
Overall efficacy	18	RCT	serious	serious	Not serious	Not serious	serious	1034	1045	RR 1.22 (1.14–1.30)	N/A	⊕○○○○○ VERY LOW
Cerebral hematoma volume	19	RCT	serious	very serious	not serious	not serious	serious	899	855	N/A	MD -6.98 (−8.76 to −5.20)	⊕○○○○○ VERY LOW
Cerebral edema volume	8	RCT	serious	very serious	not serious	not serious	serious	380	382	N/A	MD -3.67 (−5.27 to −2.06)	⊕○○○○○ VERY LOW
Activities of daily living	5	RCT	serious	very serious	not serious	not serious	N/A	278	278	N/A	SMD 2.01 (1.55–2.46)	⊕○○○○○ VERY LOW
Erythrocyte sedimentation rate	4	RCT	serious	not serious	not serious	not serious	N/A	347	347	N/A	MD 8.85 (9.56–8.14)	⊕⊕⊕○ MODERATE
Hematocrit	4	RCT	serious	not serious	not serious	serious	N/A	347	347	N/A	MD -0.4 (−1.19 to 0.39)	⊕⊕○○○○ LOW
Hypersensitive C-reactive protein	4	RCT	serious	very serious	not serious	serious	N/A	181	181	N/A	MD -8.3 (−12 to −4.61)	⊕○○○○○ VERY LOW
Low cut whole blood viscosity	4	RCT	serious	not serious	not serious	not serious	N/A	347	347	N/A	MD -3.16 (−3.59 to −2.74)	⊕⊕⊕○ MODERATE
High cut whole blood viscosity	4	RCT	serious	not serious	not serious	not serious	N/A	347	347	N/A	MD -0.83 (−0.99 to −0.67)	⊕⊕⊕○ MODERATE
Adverse events	7	RCT	serious	not serious	not serious	not serious	N/A	412	412	RR 0.63 (0.41–0.96)	N/A	⊕⊕⊕○ MODERATE

Abbreviations: No. S, number of studies; CI, confidence interval; RR, risk ratio; SMD, standardized mean differences; MD, mean differences.

## 4 Discussion

### 4.1 Interpretation of the results

#### 4.1.1 Efficacy

The results showed that for patients with ICH, compared to using CT alone, the combination of SXNI and CT may reduce the neurologic impairment scores and improve the ADL scores. In imaging aspects, it may decrease the cerebral hematoma and cerebral edema volume, alleviating brain tissue damage. In laboratory tests, it may lower ESR, hs-CRP, low cut whole blood viscosity and high cut whole blood viscosity, thereby improving patients’ hemorheological status. Therefore, these findings indicate that the combination of SXNI and CT for ICH may bring more clinical benefits to patients in terms of reducing brain injury and neurological impairment, and improving the ability to perform activities of daily living.

#### 4.1.2 Safety

The meta-analysis results of adverse events showed that there was no statistically significant difference between the SXNI combined with CT and CT alone, for patients with disease course ≤72 h or >72 h. However, the overall results indicated of adverse events in the SXNI combined with CT was lower than CT alone. Considering the paucity of studies reporting adverse events, which represented a mere fraction of the total included studies, the following conclusion was advanced with circumspection: Adding SXNI to conventional treatment may not increase the occurrence of additional safety events. And it could be further inferred that, in the treatment of patients with ICH, SXNI may demonstrates satisfactory safety.

#### 4.1.3 Subgroup analyses

According to the results of subgroup analysis, it was suggested that extending the duration of SXNI use in treating patients with disease course ≤72 h may not benefit the patients. It was noteworthy that extending the duration of SXNI use in treating patients might benefit patients in terms of reducing the cerebral hematomas volume. It was speculated that the patients’ self-healing abilities may have played a role in this effect. Increasing the dosage of SXNI may not appear to benefit patients in terms of neurological impairment score, overall efficacy, cerebral hematoma volume, ESR, HCT, low-cut whole blood viscosity, high-cut whole blood viscosity, and AE. But it may alleviate brain edema and improve prognostic outcomes. For patients with disease course >72 h of ICH, neither extending the treatment duration nor increasing the dosage of SXNI may provide benefits.

### 4.2 Heterogeneity

Among the 10 outcomes included, some exhibited high heterogeneity. To address this, subgroup analyses were conducted on all outcomes based on treatment duration and the dosage of SXNI administered. And sensitivity analysis was also performed by sequentially excluding individual studies. Through sequential exclusion of individual studies, the meta-analysis results did not show significant alterations. Therefore, the sources of high heterogeneity in outcomes such as neurological impairment score, overall efficacy, cerebral edema volume, and hs-CRP remain unclear. It is speculated that the sources of heterogeneity may stem from factors such as the severity of intracerebral hemorrhage or patients’ comorbid conditions. This speculation requires further verification.

### 4.3 Risk of bias

Due to methodological deficiencies in the included studies, there are some biases. Since none of the included studies reported allocation concealment, the risk of randomization process was judged as ‘some concerns’. Furthermore, selective reporting of results was rated as some concerns because none of the included studies reported trial registration. Overall, 93.1% of the studies were classified as some concerns regarding bias, with 6.9% classified as high risk. The assessment of publication bias indicated that four outcome measures may be subject to publication bias. This potential bias likely arises because certain types of studies (such as those with positive results, large effect sizes, or statistically significant findings) are more likely to be published, while studies with negative or null results may be overlooked or remain unpublished. Due to the methodological limitations of the included studies and potential publication bias, future high-quality studies are warranted to address these shortcomings and strengthen the current evidence base.

### 4.4 Certainty of evidence

The GRADE approach was employed to evaluate the certainty of evidence in this study. Firstly, the risk of bias was conducted on the included studies. The study found that among the overall risks of bias for all outcomes, more than two-thirds were rated as issues of concern. Consequently, the certainty of all outcomes was downgraded by one level. Secondly, in terms of inconsistency in evidence, the heterogeneity test of 5 outcomes showed that *I* ^*2*^ exceeding 75%, and the evidence was downgraded by two levels. And due to the heterogeneity test of one outcome showing I^2^ > 50% and <75%, the evidence was downgraded by one level. Thirdly, there were no significant differences in the characteristics of the included studies. Therefore, the certainty of all outcomes was not downgraded due to indirect issues. Fourthly, in terms of imprecision, the certainty of an outcome is reduced by one level because the 95% confidence interval crossed the null line. The total sample size included in all studies was less than 400 cases, therefore, the certainty of one outcome was reduced by one level. Fifthly, regarding publication bias, the funnel plots of four outcomes showed significant asymmetry, suggesting potential publication bias. Consequently, the certainty of these four outcomes was reduced by one level. Due to downgrading, the certainty of the results in this study is affected, and therefore the results should be interpreted with caution.

### 4.5 Clinical implications

The results of this study indicate that the combined use of SXNI and CT may significantly improve neurological function, promote the absorption of cerebral hematomas and cerebral edema, reduce inflammatory responses, improving microcirculatory blood flow, and demonstrated a favorable safety profile. These effects mightmay be related to the following pharmacological mechanisms of GBE: 1) Ginkgo leaf extract’s ability to regulate the inflammatory response of microglia, which in turn reduces the expression of inflammatory proteins in endothelial cells stimulated by oxidized low-density lipoprotein and decreases the release of inflammatory mediators by platelets, thereby mitigating the overall inflammatory response. And to alleviate brain damage by suppressing inflammatory responses (Sun et al., 2009; Kim et al., 2016; Wan et al., 2016; Chen et al., 2017). 2) GBE may also alleviate brain damage following cerebral hemorrhage by inhibiting neuronal apoptosis through the activation of the Akt signaling pathway (Ihl, 2013; Yu et al., 2018). 3) GBE protects human umbilical vein endothelial cells (HUVECs) from oxidized LDL-induced damage by inhibiting Akt phosphorylation, downregulating LOX-1 expression, and upregulating Sirt1 expression. Additionally, Ginkgo leaf extract may facilitate the relaxation of blood vessels, reduce blood pressure, and improve microcirculation by promoting the release of nitric oxide (NO) from endothelial cells (Kotıl et al., 2008; Wu et al., 2008; Ma et al., 2013; Silva and Martins, 2022). Under current therapeutic paradigms, Shuxuening demonstrates promising research and clinical application prospects as a potential multi-target agent for intracerebral hemorrhage management. Notably, the ≤72 h subgroup demonstrated superior therapeutic efficacy in cerebral edema reduction compared to the >72 h subgroup. Moreover, extended treatment duration or increased SXNI dosage may potentially enhance anti-inflammatory effects. Speculatively, this finding may be attributed to the following mechanisms*: 1)* The peak disruption of the blood-brain barrier (BBB) occurs within hours of ICH onset (Li Z. et al., 2020). *Ginkgolides—the primary active components of SXNI—potently inhibit MMP-9 activation, a pivotal protease mediating early BBB damage* (Fang et al., 2015). *Early administration might therefore maximize cerebroprotective effects through BBB stabilization. 2)* It might be associated with drug accumulation effects or exhibit a dose-dependent manner (Chen et al., 2022). Although the quality of evidence was low, this study still provides some references for the clinical use of SXNI in the treatment of cerebral hemorrhage, and provides ideas for further clinical research.

### 4.6 Strengths and limitations

This study categorized patients into two phases: patients with disease course ≤72 h or >72h, and conducted a comprehensive evaluation of the efficacy and safety of SXNI across three aspects: clinical manifestations, imaging examinations, and laboratory tests. To explore the impact of various factors on treatment, the study also conducted subgroup analyses based on treatment duration and SXNI dosage. And discussions and speculations were made regarding the results of subgroup analyses.

This study has several limitations. First, the literature quality incorporated in this study was low, and the outcomes are susceptible to bias, diminishing the evidence’s credibility. Secondly, among the 29 literature included in this study, only literature from China and no literature from other countries were included, and all the included trials were conducted in China. This reduces the applicability of the evidence to other countries or regions. Third, the literature included in this study lacks follow-up and reports of end points, which may only represent the safety and efficacy of SXNI in the short term. Fourthly, due to the limited covariate information reported in the included literature, various subgroup analyses were conducted, yet the sources of significant heterogeneity in the results remain unclear.

### 4.7 Future perspectives

The scarcity of well-conducted clinical trials is a prevalent concern within ethnopharmacology studies (Liu et al., 2024a). High-quality randomized controlled trials that involve substantial participant numbers and extended follow-up periods, characterized by meticulous design and execution, tend to demand greater financial and temporal investments, potentially accounting for the dearth of high-quality trial in included studies. To enhance the certainty of evidence regarding SXNI in cerebral hemorrhage, high-quality, large-sample, multicenter randomized controlled trials should be conducted to evaluate the efficacy and safety of SXNI in the treatment of cerebral hemorrhage. In the trial design, it is recommended to include outcomes that reflect long-term efficacy, such as mortality rates. It is also advised to use standardized assessment tools and methods, such as the NIHSS, to reflect specific neurological performance. And the pharmacological mechanisms of SXNI in the treatment of ICH still require further investigation.

## 5 Conclusion

This study suggests that, compared with CT alone, the combined use of SXNI and CT may improve neurological function. Additionally, it may promotes hematoma absorption, reduces inflammatory responses, and does not increase any additional safety events. This indicates that the efficacy and safety of SXNI in the treatment of ICH may be favorable. Given that the certainty of the evidence ranges from moderate to very low, it is necessary to conduct high-quality randomized controlled trials with large sample sizes to verify these findings.

## Data Availability

The original contributions presented in the study are included in the article/Supplementary Material, further inquiries can be directed to the corresponding authors.
