# Supplementary material for: Efficacy and safety of Shuxuening injection in intracerebral hemorrhage: a systematic review and meta-analysis
Source: Front Pharmacol. 2025 May 19;16:1537679. doi: 10.3389/fphar.2025.1537679 (PMC12127381; doi:10.3389/fphar.2025.1537679)
Supplement: Supplementary file 3 [file DataSheet3.pdf]

Supplementary Material

1 Detailed information for Shuxuening injection

Shuxuening injection is a sterilized aqueous solution made of *ginkgo biloba* extract.The details are shown in Table 1.

Table 1 Detailed information for Shuxuening injection

Type	Ingredients	Specifications	Usage and dosage	Indication
Shuxuening injection	*Ginkgo biloba extract.The main components are total flavonol glycosides and ginkgolide*	2ml: 7.0mg<br>5ml: 17.5mg	Intravenous drip, 5-20ml at a time, diluted in 250-500ml of 5-10% glucose injection and administered slowly. 1-2 times a day.	Dilated blood vessels and improved microcirculation. Used for ischemic cardiovascular and cerebrovascular diseases, coronary heart disease, angina pectoris, cerebral embolism, cerebral vasospasm, etc.

2 Search Queries

The search queries for China National Knowledge Infrastructure Database (CNKI), VIP Database for Chinese Technical Periodicals (VIP), Wanfang Database (Wanfang), Chinese Biomedical Literature Database (SinoMed), Web of Science, Pubmed, Embase, and Cochrane Library are shown in Table 2.

Table 2 Search queries for each database

Database	Search query
CNKI	(TKA = '中风' OR TKA = '脑溢血' OR TKA = '脑出血' OR TKA = '卒中' OR TKA = '脑血管意外' OR TKA = '脑血管破裂') AND (TKA = '舒血宁' OR TKA = '银杏叶提取物') AND FT = '随机'
VIP	M = (中风 OR 脑溢血 OR 脑出血 OR 卒中 OR 脑血管意外 OR 脑血管破裂) AND M = (舒血宁 OR 银杏叶提取物) AND U = 随机
Wanfang	主题:(中风 or 脑溢血 or 脑出血 or 卒中 or 脑血管意外 or 脑血管破裂) and 主题:(舒血宁 or 银杏叶提取物) and 全部:(随机)
SinoMed	("随机"[全部字段:智能]) AND ("舒血宁"[常用字段:智能] OR "银杏叶提取物"[常用字段:智能]) AND ("中风"[常用字段:智能] OR "脑溢血"[常用字段:智能] OR "脑出血"[常用字段:智能] OR "卒中"[常用字段:智能] OR "脑血管意外"[常用字段:智能] OR "脑血管破裂"[常用字段:智能])

Web of Science	#1 TS=(Cerebral Hemorrhage OR Hemorrhage, Cerebrum OR Cerebrum Hemorrhage OR Cerebrum Hemorrhages OR Hemorrhages, Cerebrum OR Cerebral Parenchymal Hemorrhage OR Cerebral Parenchymal Hemorrhages OR Hemorrhage, Cerebral Parenchymal OR Hemorrhages, Cerebral Parenchymal OR Parenchymal Hemorrhage, Cerebral OR Parenchymal Hemorrhages, Cerebral OR Intracerebral Hemorrhage OR Hemorrhage, Intracerebral OR Hemorrhages, Intracerebral OR Intracerebral Hemorrhages OR Hemorrhage, Cerebral OR Cerebral Hemorrhages OR Hemorrhages, Cerebral OR Brain Hemorrhage, Cerebral OR Brain Hemorrhages, Cerebral OR Cerebral Brain Hemorrhage OR Cerebral Brain Hemorrhages OR Hemorrhage, Cerebral Brain OR Hemorrhages, Cerebral Brain)<br>#2 TS=(*ginkgo biloba* OR *Ginkgo* OR *Ginkgo biloba* OR Yinxing OR Yinxingye OR Shuxuening OR Ginaton)<br>#3 (ALL=(randomized) OR TS=(RCT))<br>#4=#1 AND #2 AND #3
Pumbed	#1 "Cerebral Hemorrhage[Mesh]" OR "Hemorrhage, Cerebrum[Title/Abstract]" OR "Cerebrum Hemorrhage[Title/Abstract]" OR "Cerebrum Hemorrhages[Title/Abstract]" OR "Hemorrhages, Cerebrum[Title/Abstract]" OR "Cerebral Parenchymal Hemorrhage[Title/Abstract]" OR "Cerebral Parenchymal Hemorrhages[Title/Abstract]" OR "Hemorrhage, Cerebral Parenchymal[Title/Abstract]" OR "Hemorrhages, Cerebral Parenchymal[Title/Abstract]" OR "Parenchymal Hemorrhage, Cerebral[Title/Abstract]" OR "Parenchymal Hemorrhages, Cerebral[Title/Abstract]" OR "Intracerebral Hemorrhage[Title/Abstract]" OR "Hemorrhage, Intracerebral[Title/Abstract]" OR "Hemorrhages, Intracerebral[Title/Abstract]" OR "Intracerebral Hemorrhages[Title/Abstract]" OR "Hemorrhage, Cerebral[Title/Abstract]" OR "Cerebral Hemorrhages[Title/Abstract]" OR "Hemorrhages, Cerebral[Title/Abstract]" OR "Brain Hemorrhage, Cerebral[Title/Abstract]" OR "Brain Hemorrhages, Cerebral[Title/Abstract]" OR "Cerebral Brain Hemorrhage[Title/Abstract]" OR "Cerebral Brain Hemorrhage Cerebral Brain Hemorrhages[Title/Abstract]" OR "Hemorrhage, Cerebral Brain[Title/Abstract]" OR "Hemorrhages, Cerebral Brain[Title/Abstract]"<br>#2 "*ginkgo biloba*"[MeSH Terms] OR "*Ginkgo*"[Title/Abstract] OR "*Ginkgo biloba*"[Title/Abstract] OR "Yinxing"[Title/Abstract] OR "Yinxingye"[Title/Abstract] OR "Shuxuening"[Title/Abstract] OR "Ginaton"[Title/Abstract]<br>#3 Clinical Trial[Publication Type] OR randomized controlled study[Title/Abstract] OR randomized controlled trial[Title/Abstract] OR randomized study[Title/Abstract] OR randomized trial[Title/Abstract] OR randomized placebo-controlled study[Title/Abstract] OR randomized placebo-controlled trial[Title/Abstract] OR randomized placebo controlled[Title/Abstract] OR randomized placebo-controlled[Title/Abstract] OR randomized double-blind[Title/Abstract] OR randomized double

	blin[Title/Abstract]<br>#4 #1 AND #2 AND #3
Embase	#1 'hemorrhage, cerebrum':ti,ab,kw OR 'cerebrum hemorrhage':ti,ab,kw OR 'cerebrum hemorrhages':ti,ab,kw OR 'hemorrhages, cerebrum':ti,ab,kw OR 'cerebral parenchymal hemorrhage':ti,ab,kw OR 'cerebral parenchymal hemorrhages':ti,ab,kw OR 'hemorrhage, cerebral parenchymal':ti,ab,kw OR 'hemorrhages, cerebral parenchymal':ti,ab,kw OR 'parenchymal hemorrhage, cerebral':ti,ab,kw OR 'parenchymal hemorrhages, cerebral':ti,ab,kw OR 'intracerebral hemorrhage':ti,ab,kw OR 'hemorrhage, intracerebral':ti,ab,kw OR 'hemorrhages, intracerebral':ti,ab,kw OR 'intracerebral hemorrhages':ti,ab,kw OR 'hemorrhage, cerebral':ti,ab,kw OR 'cerebral hemorrhages':ti,ab,kw OR 'hemorrhages, cerebral':ti,ab,kw OR 'brain hemorrhage, cerebral':ti,ab,kw OR 'brain hemorrhages, cerebral':ti,ab,kw OR 'cerebral brain hemorrhage':ti,ab,kw OR 'cerebral brain hemorrhages':ti,ab,kw OR 'hemorrhage, cerebral brain':ti,ab,kw OR 'hemorrhages, cerebral brain':ti,ab,kw<br>#2 'brain hemorrhage'/exp<br>#3=#1 OR #2<br>#4 '*ginkgo biloba* extract'/exp<br>#5 *ginkgo*:ti,ab,kw OR '*ginkgo biloba*':ti,ab,kw OR yinxing:ti,ab,kw OR yinxingye:ti,ab,kw OR shuxuening:ti,ab,kw OR ginaton:ti,ab,kw<br>#6= #4 OR #5<br>#7 'randomized controlled trial'/exp<br>#8 'clinical trial':ti,ab,kw OR 'randomized controlled study':ti,ab,kw OR 'randomized controlled trial':ti,ab,kw OR 'randomized study':ti,ab,kw OR 'randomized trial':ti,ab,kw OR 'randomized placebo-controlled study':ti,ab,kw OR 'randomized placebo-controlled trial':ti,ab,kw OR 'randomized placebo controlled':ti,ab,kw OR 'randomized placebo-controlled':ti,ab,kw OR 'randomized double-blind':ti,ab,kw OR 'randomized double blin':ti,ab,kw<br>#9=#7 OR #8<br>#10=#3 AND #6 AND #9

Cochrane Library	#1 MeSH descriptor: [Cerebral Hemorrhage] explode all trees<br>#2 (Hemorrhage, Cerebrum OR Cerebrum Hemorrhage OR Cerebrum Hemorrhages OR Hemorrhages, Cerebrum OR Cerebral Parenchymal Hemorrhage OR Cerebral Parenchymal Hemorrhages OR Hemorrhage, Cerebral Parenchymal OR Hemorrhages, Cerebral Parenchymal OR Parenchymal Hemorrhage, Cerebral OR Parenchymal Hemorrhages, Cerebral OR Intracerebral Hemorrhage OR Hemorrhage, Intracerebral OR Hemorrhages, Intracerebral OR Intracerebral Hemorrhages OR Hemorrhage, Cerebral OR Cerebral Hemorrhages OR Hemorrhages, Cerebral OR Brain Hemorrhage, Cerebral OR Brain Hemorrhages, Cerebral OR Cerebral Brain Hemorrhage OR Cerebral Brain Hemorrhages OR Hemorrhage, Cerebral Brain OR Hemorrhages, Cerebral Brain):ti,ab,kw<br>#3=#1 OR #2<br>#4 MeSH descriptor: [*Ginkgo biloba*] explode all trees<br>#5 (*ginkgo biloba* OR *Ginkgo* OR *Ginkgo biloba* OR Yinxing OR Yinxingye OR Shuxuening OR Ginaton):ti,ab,kw<br>#6=#4 OR #5<br>#7 MeSH descriptor: [Randomized Controlled Trial] explode all trees<br>#8 (Clinical Trial OR randomized controlled study OR randomized controlled trial OR randomized study OR randomized trial OR randomized placebo-controlled study OR randomized placebo-controlled trial OR randomized placebo controlled OR randomized placebo-controlled OR randomized double-blind OR randomized double blin):ti,ab,kw<br>#9=#7 OR #8<br>#10=#3 AND #6 AND #9

Abbreviation: CNKI, China National Knowledge Infrastructure Database, VIP, VIP Database for Chinese Technical Periodicals, Wanfang, Wanfang Database, SinoMed, Chinese Biomedical Literature Database

3 Diagnostic criteria for intracerebral haemorrhage

Diagnostic criteria: (1) Acute onset; (2) Focal neurologic deficit symptoms (a minority with global neurologic deficit), often accompanied by headache, vomiting, elevated blood pressure, and varying degrees of consciousness disturbance; (3) Cerebral CT or MRI showing hemorrhagic lesions; (4) Exclusion of non-vascular brain causes.

4 Criteria of the overall efficacy

The present study assessed the overall therapeutic efficacy based on the changes in the National Institutes of Health Stroke Scale (NIHSS) scores before and after treatment. The specific criteria for judgment are as follows:

Effective: A reduction of ≥18% in the neurofunctional impairment score after treatment.

Ineffective: A reduction of < 18% in the neurofunctional impairment score after treatment.

5 The measurement of the image data of the included studies

Measurement of cerebral hematoma volume: The cerebral hematoma volume was calculated using the Tada formula based on CT scans, as follows: Hematoma volume (mL) = length (cm) × width (cm) × number of slices × π/6 on the slice with the largest hematoma. The changes in hematoma volume were compared between the two groups of patients.

Measurement of cerebral edema volume: The cerebral edema volume (mL) was calculated using CT scans with the following formula:

Edema volume (mL) = [Length (cm) × Width (cm) × Number of slices × π/6] (for the largest slice of edema + hematoma) - [Length (cm) × Width (cm) × Number of slices × π/6] (for the largest slice of hematoma alone).

(Note: This formula estimates edema volume by subtracting the hematoma volume from the combined volume of edema and hematoma.)

6 Rules for Assessment of the Certainty of Evidence

The assessment of certainty of evidence was divided into five elements: study design, inconsistency, indirectness, imprecision, and publication bias, each with their respective criteria for downgrade as shown in Table 3.

Table 3 Rules for assessment of the certainty of evidence

Element	Criteria for downgrade
Risk of bias	If the majority of the information based on the revised Cochrane Risk of Bias tool assessment was rated as moderate, the evidence was downgraded by one level. If the majority was rated as high, the evidence was downgraded by two levels.
Inconsistency	If heterogeneity tests showed I^2 exceeding 75%, the evidence was downgraded by two levels. If I^2 was exceeding 50% and less than 75%, the evidence was downgraded by one level.
Indirectness	Assessment included several components: Population differences, Intervention differences, Outcome measurement differences, and Indirect comparisons. If there was a serious suspicion regarding the directness of the evidence, the evidence was downgraded by one level. If there was a very serious suspicion regarding the directness of the evidence, the evidence was downgraded by two levels.
Imprecision	If the 95% confidence interval crossed the null line, the evidence was downgraded by one level. With a total sample size of less than 300 for categorical variables and less than 400 for continuous variables included in all studies, the evidence was downgraded by one level.

Publication bias	If there was publication bias, the evidence was downgraded by one level.

7 Sensitivity analysis

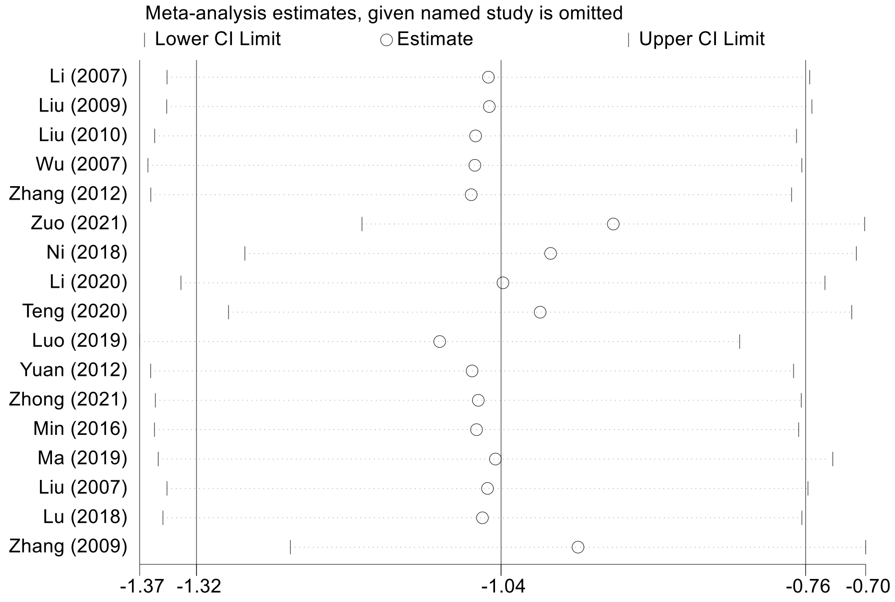

Figure 1 The results of the sensitivity analysis of the effect of neurological impairment score.

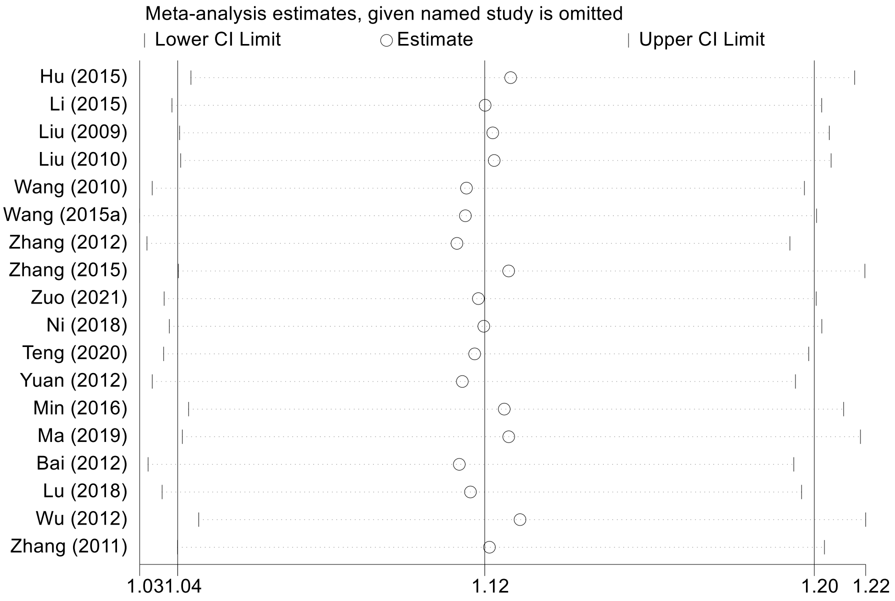

Figure 2 The results of the sensitivity analysis of the effect of overall efficacy.

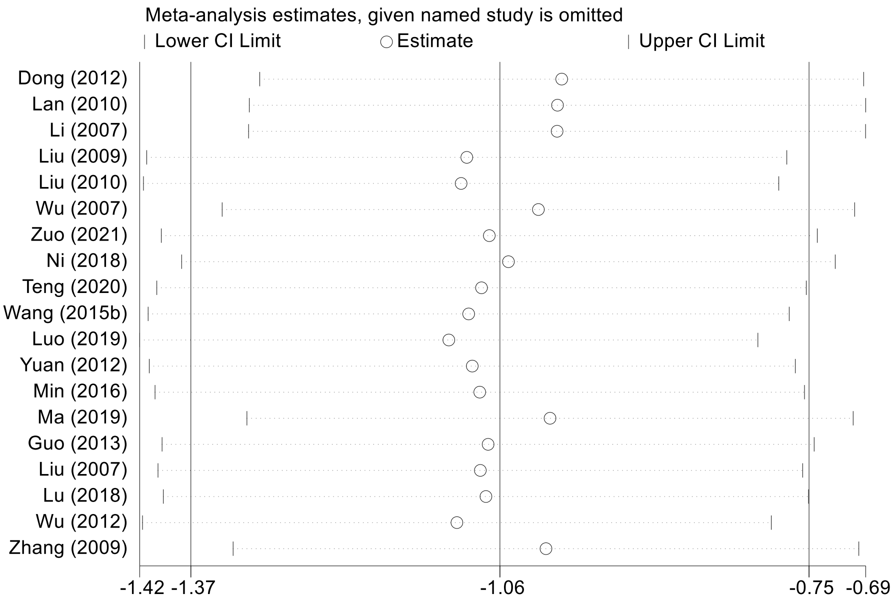

Figure 3 The results of the sensitivity analysis of the effect of cerebral hematoma volume.

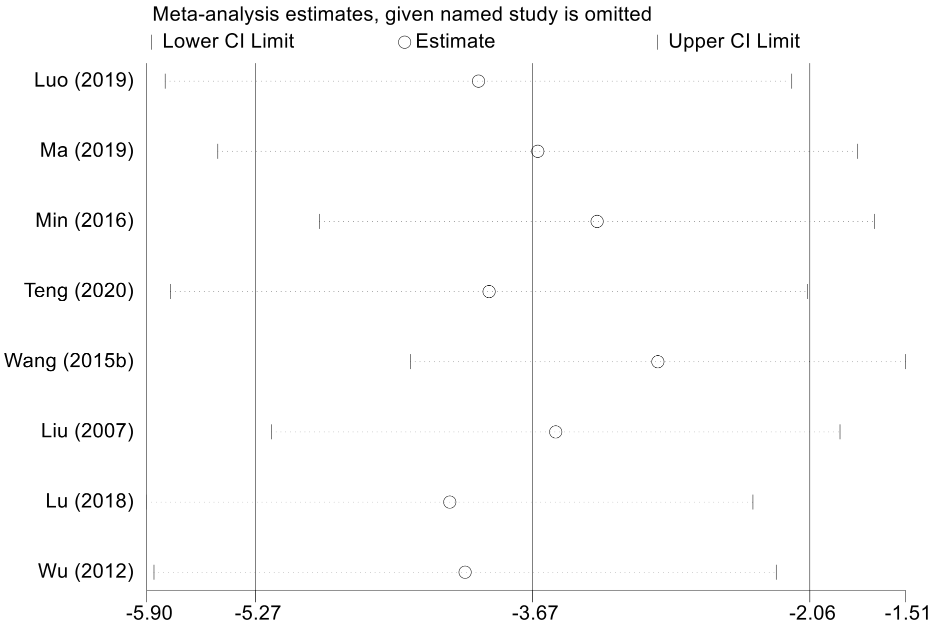

Figure 4 The results of the sensitivity analysis of the effect of cerebral edema volume.

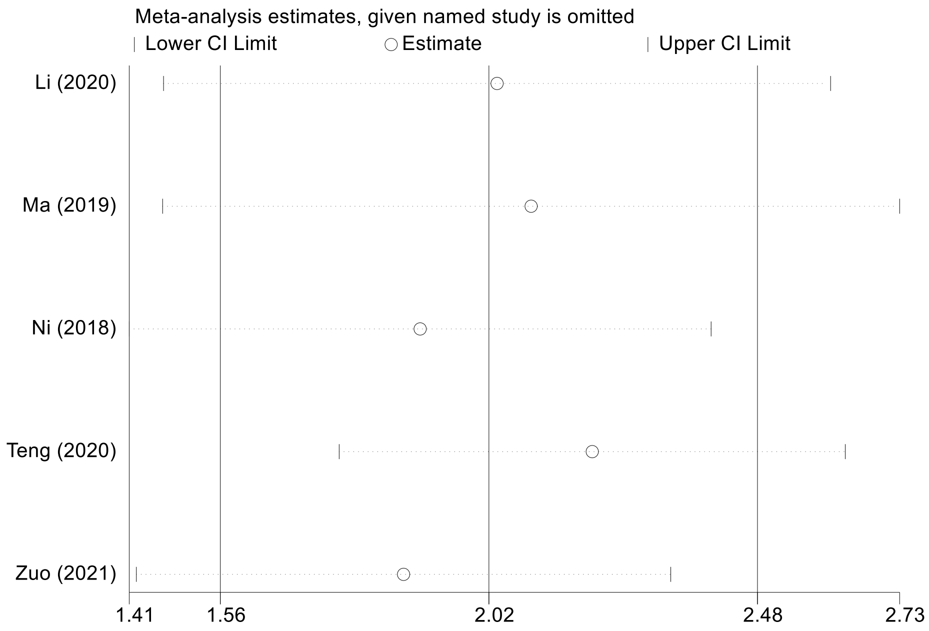

Figure 5 The results of the sensitivity analysis of the effect of ADL.

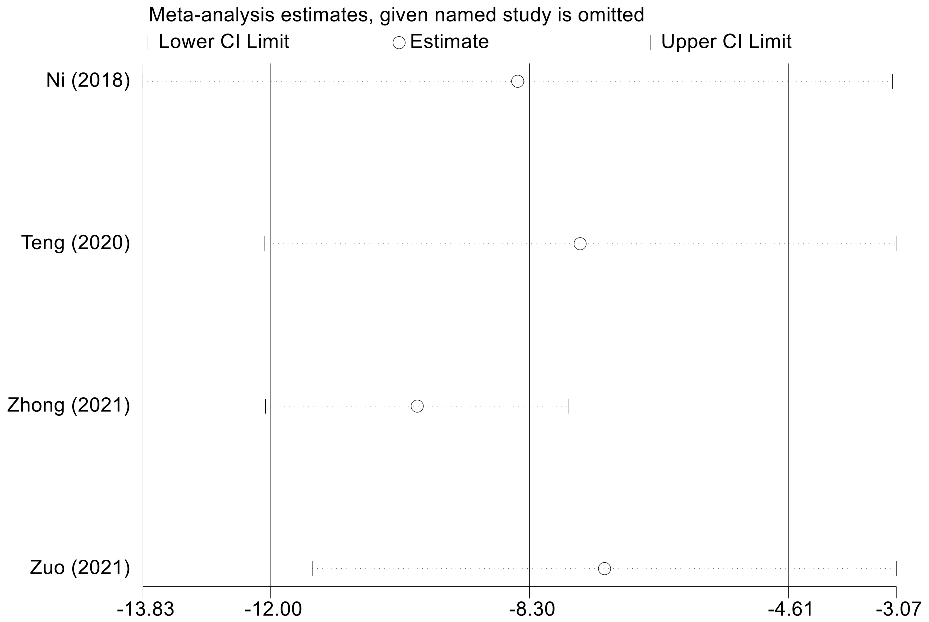

Figure 6 The results of the sensitivity analysis of the effect of hs-CRP.

8 Abbreviations

Table 4 Abbreviations

Abbreviations	
(ADL)	activities of daily living
(AE)	adverse events
(CI)	confidence interval
(CNKI)	China National Knowledge Infrastructure Database

(CSS)	China Stroke Scale
(CT)	conventional treatment
(DALY)	disability-adjusted life-year
(ESR)	erythrocyte sedimentation rate
(ESS)	European Stroke Scale
(GBE)	Ginkgo Biloba extract
(HCT)	hematocrit
(HE)	hematoma expansion
(hs-CRP)	hypersensitive C-reactive protein
(ICAM-1)	intercellular adhesion molecule 1
(ICH)	intracerebral hemorrhage
(LOX-1)	low-density lipoprotein receptor-1
(MD)	mean differences
(NF-κB)	nuclear factor κB
(NIHSS)	National Institute of Health Stroke Scale
(NO)	nitric oxide
(PRISMA, 2020)	Preferred Reporting Items for Systematic Reviews and Meta-Analyses 2020
(RCT)	randomized controlled trial
(ROB 2.0)	the revised Cochrane risk of bias tool for randomized trials 2.0
(RR)	risk ratios
(SinoMed)	Chinese Biomedical Literature Database
(Sirt1)	Sirtuin 1
(SMD)	standardized mean differences
(SXNI)	Shuxuening injection
(TLR4)	toll-like receptor 4
(VCAM-1)	vascular cell adhesion molecule 1
(VIP)	VIP Database for Chinese Technical Periodicals
(Wanfang)	Wanfang Database

9 The information on Shuxuening injection from the original literature

The information for each original literature on Shuxuening injection is presented in Table 5.

Table 5 The information on Shuxuening injection from the original literature.

Study	Formulation	Source	Species	Quality control reported? (Y/N)	Chemical analysis reported? (Y/N)
（Bai and Wang, 2012）	Shuxuening injection	Shanxi Yinhu Pharmaceutical Co., Ltd	*Ginkgo biloba extract.The main components are total flavonol glycosides and*	N	N

			ginkgolide		
(Dong, 2012)	Shuxuening injection	Heilongjiang Zhenbao Island Pharmaceutical Co., Ltd	*Ginkgo biloba extract.The main components are total flavonol glycosides and ginkgolide*	N	N
(Guo, 2013)	Shuxuening injection	-	*Ginkgo biloba extract.The main components are total flavonol glycosides and ginkgolide*	N	N
(Hu and Pu, 2015)	Shuxuening injection	Shenwei Pharmaceutical Group Co., Ltd	*Ginkgo biloba extract.The main components are total flavonol glycosides and ginkgolide*	N	N
(Lan et al., 2010)	Shuxuening injection	-	*Ginkgo biloba extract.The main components are total flavonol glycosides and ginkgolide*	N	N
(Li and Hu, 2015)	Shuxuening injection	-	*Ginkgo biloba extract.The main components are total flavonol glycosides and ginkgolide*	N	N
(Li et al., 2007)	Shuxuening injection	Sanjiu Wanrong Pharmaceutical Co., Ltd	*Ginkgo biloba extract.The main components are total flavonol glycosides and ginkgolide*	N	N
(Li,	Shuxuening	Taiwan	*Ginkgo biloba*	N	N

2020)	injection	Jisheng Chemical Pharmaceutical Co., Ltd	*extract.The main components are total flavonol glycosides and ginkgolide*		
(Liu and Liu, 2009)	Shuxuening injection	Shenwei Pharmaceutical Group Co., Ltd	*Ginkgo biloba extract.The main components are total flavonol glycosides and ginkgolide*	N	N
(Liu et al., 2010)	Shuxuening injection	Shenwei Pharmaceutical Group Co., Ltd	*Ginkgo biloba extract.The main components are total flavonol glycosides and ginkgolide*	N	N
(Liu, 2007)	Shuxuening injection	-	*Ginkgo biloba extract.The main components are total flavonol glycosides and ginkgolide*	N	N
(Lu et al., 2018)	Shuxuening injection	Beijing Huarun High Tech Natural Medicine Co., Ltd	*Ginkgo biloba extract.The main components are total flavonol glycosides and ginkgolide*	N	N
(Luo and Liu, 2019)	Shuxuening injection	-	*Ginkgo biloba extract.The main components are total flavonol glycosides and ginkgolide*	N	N
(Ma and Wang,	Shuxuening injection	Yuekang Pharmaceutical Group	*Ginkgo biloba extract.The main*	N	N

2019)		Co., Ltd	*components are total flavonol glycosides and ginkgolide*		
(Min et al., 2016)	Shuxuening injection	Yuekang Pharmaceutical Group Co., Ltd	*Ginkgo biloba extract.The main components are total flavonol glycosides and ginkgolide*	N	N
(Ni et al., 2018)	Shuxuening injection	Taiwan Jisheng Chemical Pharmaceutical Co., Ltd	*Ginkgo biloba extract.The main components are total flavonol glycosides and ginkgolide*	N	N
(Teng, 2020)	Shuxuening injection	Yuekang Pharmaceutical Group Co., Ltd	*Ginkgo biloba extract.The main components are total flavonol glycosides and ginkgolide*	N	N
(Wang and Wang, 2010)	Shuxuening injection	-	*Ginkgo biloba extract.The main components are total flavonol glycosides and ginkgolide*	N	N
(Wang et al., 2015)	Shuxuening injection	Beijing Huarun High Tech Natural Medicine Co., Ltd	*Ginkgo biloba extract.The main components are total flavonol glycosides and ginkgolide*	N	N
(Wang, 2015)	Shuxuening injection	Shanxi Zhendong Taisheng Pharmaceutical Co., Ltd	*Ginkgo biloba extract.The main components are total flavonol*	N	N

			glycosides and ginkgolide		
(Wu, 2007)	Shuxuening injection	Sanjiu Wanrong Pharmaceutical Co., Ltd	*Ginkgo biloba extract.The main components are total flavonol glycosides and ginkgolide*	N	N
(Wu, 2012)	Shuxuening injection	Shenwei Pharmaceutical Group Co., Ltd	*Ginkgo biloba extract.The main components are total flavonol glycosides and ginkgolide*	N	N
(Yuan et al., 2012)	Shuxuening injection	-	*Ginkgo biloba extract.The main components are total flavonol glycosides and ginkgolide*	N	N
(Zhang et al., 2012)	Shuxuening injection	-	*Ginkgo biloba extract.The main components are total flavonol glycosides and ginkgolide*	N	N
(Zhang et al., 2015)	Shuxuening injection	Heilongjiang Zhenbao Island Pharmaceutical Co., Ltd	*Ginkgo biloba extract.The main components are total flavonol glycosides and ginkgolide*	N	N
(Zhang, 2009)	Shuxuening injection	-	*Ginkgo biloba extract.The main components are total flavonol glycosides and ginkgolide*	N	N

(Zhang, 2011)	Shuxuening injection	-	*Ginkgo biloba extract.The main components are total flavonol glycosides and ginkgolide*	N	N
(Zhong et al., 2021)	Shuxuening injection	Yuekang Pharmaceutical Group Co., Ltd	*Ginkgo biloba extract.The main components are total flavonol glycosides and ginkgolide*	N	N
(Zuo et al., 2021)	Shuxuening injection	-	*Ginkgo biloba extract.The main components are total flavonol glycosides and ginkgolide*	N	N
